# Comparison of artemether-lumefantrine and chloroquine with and without primaquine for the treatment of *Plasmodium vivax* infection in Ethiopia: A randomized controlled trial

**DOI:** 10.1371/journal.pmed.1002299

**Published:** 2017-05-16

**Authors:** Tesfay Abreha, Jimee Hwang, Kamala Thriemer, Yehualashet Tadesse, Samuel Girma, Zenebe Melaku, Ashenafi Assef, Moges Kassa, Mark D. Chatfield, Keren Z. Landman, Stella M. Chenet, Naomi W. Lucchi, Venkatachalam Udhayakumar, Zhiyong Zhou, Ya Ping Shi, S. Patrick Kachur, Daddi Jima, Amha Kebede, Hiwot Solomon, Addis Mekasha, Bereket Hailegiorgis Alemayehu, Joseph L. Malone, Gunewardena Dissanayake, Hiwot Teka, Sarah Auburn, Lorenz von Seidlein, Ric N. Price

**Affiliations:** 1 ICAP, Columbia University Mailman School of Public Health, Addis Ababa, Ethiopia; 2 US President’s Malaria Initiative, Malaria Branch, Division of Parasitic Diseases and Malaria, US Centers for Disease Control and Prevention, Atlanta, Georgia, United States of America; 3 Global Health Group, University of California San Francisco, San Francisco, California, United States of America; 4 Global and Tropical Health Division, Menzies School of Health Research, Charles Darwin University, Darwin, Northern Territory, Australia; 5 Ethiopian Public Health Institute, Addis Ababa, Ethiopia; 6 Malaria Branch, Division of Parasitic Diseases and Malaria, US Centers for Disease Control and Prevention, Atlanta, Georgia, United States of America; 7 Federal Ministry of Health, Addis Ababa, Ethiopia; 8 Oromia Regional Health Bureau, Addis Ababa, Ethiopia; 9 US President’s Malaria Initiative, US Agency for International Development, Addis Ababa, Ethiopia; 10 Mahidol Oxford Tropical Medicine Research Unit, Mahidol University, Bangkok, Thailand; 11 Centre for Tropical Medicine and Global Health, Nuffield Department of Medicine, University of Oxford, Oxford, United Kingdom; ISGlobal, SPAIN

## Abstract

**Background:**

Recent efforts in malaria control have resulted in great gains in reducing the burden of *Plasmodium falciparum*, but *P*. *vivax* has been more refractory. Its ability to form dormant liver stages confounds control and elimination efforts. To compare the efficacy and safety of primaquine regimens for radical cure, we undertook a randomized controlled trial in Ethiopia.

**Methods and findings:**

Patients with normal glucose-6-phosphate dehydrogenase status with symptomatic *P*. *vivax* mono-infection were enrolled and randomly assigned to receive either chloroquine (CQ) or artemether-lumefantrine (AL), alone or in combination with 14 d of semi-supervised primaquine (PQ) (3.5 mg/kg total). A total of 398 patients (*n* = 104 in the CQ arm, *n* = 100 in the AL arm, *n* = 102 in the CQ+PQ arm, and *n* = 92 in the AL+PQ arm) were followed for 1 y, and recurrent episodes were treated with the same treatment allocated at enrolment. The primary endpoints were the risk of *P*. *vivax* recurrence at day 28 and at day 42.

The risk of recurrent *P*. *vivax* infection at day 28 was 4.0% (95% CI 1.5%–10.4%) after CQ treatment and 0% (95% CI 0%–4.0%) after CQ+PQ. The corresponding risks were 12.0% (95% CI 6.8%–20.6%) following AL alone and 2.3% (95% CI 0.6%–9.0%) following AL+PQ. On day 42, the risk was 18.7% (95% CI 12.2%–28.0%) after CQ, 1.2% (95% CI 0.2%–8.0%) after CQ+PQ, 29.9% (95% CI 21.6%–40.5%) after AL, and 5.9% (95% CI 2.4%–13.5%) after AL+PQ (overall *p <* 0.001). In those not prescribed PQ, the risk of recurrence by day 42 appeared greater following AL treatment than CQ treatment (HR = 1.8 [95% CI 1.0–3.2]; *p* = 0.059). At the end of follow-up, the incidence rate of *P*. *vivax* was 2.2 episodes/person-year for patients treated with CQ compared to 0.4 for patients treated with CQ+PQ (rate ratio: 5.1 [95% CI 2.9–9.1]; *p <* 0.001) and 2.3 episodes/person-year for AL compared to 0.5 for AL+PQ (rate ratio: 6.4 [95% CI 3.6–11.3]; *p <* 0.001). There was no difference in the occurrence of adverse events between treatment arms.

The main limitations of the study were the early termination of the trial and the omission of haemoglobin measurement after day 42, resulting in an inability to estimate the cumulative risk of anaemia.

**Conclusions:**

Despite evidence of CQ-resistant *P*. *vivax*, the risk of recurrence in this study was greater following treatment with AL unless it was combined with a supervised course of PQ. PQ combined with either CQ or AL was well tolerated and reduced recurrence of vivax malaria by 5-fold at 1 y.

**Trial registration:**

ClinicalTrials.gov NCT01680406

## Abstract 2

Kamala Thriemer and colleagues report on a randomized controlled trial of treatments for Plasmodium vivax malaria.

## Author summary

**Why was this study done?:**

- In areas where *Plasmodium vivax* is endemic, recurrent parasitaemia arises from chloroquine (CQ) resistance and the reactivation of dormant hypnozoites. Recurrent parasitaemia is associated with significant morbidity and mortality. Providing safe and effective radical cure of blood and liver stages of the parasite is critical for the control and elimination of *P*. *vivax* malaria.
- Two systematic reviews of *P*. *vivax* clinical trials highlighted a declining efficacy for CQ in many *P*. *vivax* endemic areas and marked heterogeneity in study design for determining primaquine (PQ) efficacy. The conclusions of these reviews were that clinical trials to determine the efficacy of radical cure should follow up patients for a prolonged period to capture late relapses and should quantify the incidence rate of multiple recurrences, not just the time to the first recurrence.
- The rise in CQ-resistant *P*. *vivax* provides a good rationale for artemisinin combination therapy for *P*. *vivax* malaria; however, the efficacy of artemisinin combination therapies in combination with PQ radical cure is poorly documented.

**What did the researchers do and find?:**

- We randomized patients to four treatment arms: one group received CQ only (the current recommended treatment for *P*. *vivax* malaria in Ethiopia), the second group received CQ plus PQ, the third group received artemether-lumefantrine (AL) alone, and the fourth group received AL plus PQ. All patients were followed up for a year and were treated with the same treatment for every *P*. *vivax* malaria episode.
- We quantified the risk of *P*. *vivax* infections at day 28 after treatment and also over 12 months. The risk of recurrence by day 28 and 42 was greater following AL than CQ. The addition of PQ to either CQ or AL reduced the risk of recurrence 3-fold by day 42, and 2- to 3-fold over one year.
- Patients treated with PQ had on average only 0.5 *P*. *vivax* malaria episodes per year, whereas patients not treated with PQ had on average two episodes per year. The efficacy of PQ treatment for recurrences, which was unsupervised, was 3- to 4-fold lower than that of the initial treatment, which was semi-supervised.

**What do these findings mean?:**

- In Ethiopia there is evidence of CQ resistance; nevertheless, in this study CQ monotherapy had greater efficacy than AL therapy at day 42.
- The addition of PQ radical cure to either CQ or AL provided major benefits in reducing subsequent recurrent infection.
- PQ radical cure should be included in the treatment schedule in Ethiopia and other areas with high relapse risk to reduce relapsing infection and transmission, but further work is needed to improve adherence to the current 14-day regimen to ensure maximum public health impact.

## Introduction

Almost 3 billion people live at risk of *Plasmodium vivax* infection [1,2], with over 100 million clinical malaria cases estimated to occur each year [3]. The greatest burden of *P*. *vivax* malaria is in the Asia-Pacific region and South America, whereas on the African continent, *P*. *vivax* infection is limited mostly to the Horn of Africa. Recent intensification of malaria control efforts has reduced the global burden of *P*. *falciparum* malaria, but *P*. *vivax* has been more refractory. Vivax malaria is more difficult to cure than falciparum malaria, due to its ability to form dormant liver stages (hypnozoites) that reactivate periodically, causing relapsing infections and onward transmission. Chloroquine (CQ) remains the mainstay of treatment for vivax malaria in most endemic countries, but drug resistance has emerged in South-East Asia and is spreading [4]. Relapsing and increasingly frequent recrudescent infections cause repeated symptomatic illnesses, worsening the risk of anaemia and severe and fatal disease [5,6].

Although *P*. *vivax* is rare on the African continent outside the Horn of Africa, in Ethiopia it is responsible for approximately 40% of all clinical malaria [7–9]. National treatment guidelines recommend CQ as first-line treatment for uncomplicated *P*. *vivax* malaria. Artemether-lumefantrine (AL) is used widely for mixed-species infections of *P*. *falciparum* and *P*. *vivax*, and for cases of clinical malaria where diagnostics to determine the *Plasmodium* species are unavailable. Both CQ and AL have schizonticidal efficacy but lack activity against the liver stages. Primaquine (PQ), an 8-aminoquinoline, is the only currently available hypnozoiticide, but can cause severe haemolysis in glucose-6-phosphate-dehydrogenase (G6PD)–deficient patients; for this reason, programmes are often reluctant to recommend PQ, and healthcare providers are hesitant to prescribe it. Ethiopia has embarked on an ambitious malaria control programme, supporting the country’s Health Sector Development Plan as well as the national child survival strategy. The control and elimination of malaria will require deployment of a safe and effective radical cure of *P*. *vivax*. At the time this study was conducted, PQ radical cure was not part of the national antimalarial guidelines for patients living in malaria endemic areas of the country. To compare suitable treatment strategies, we undertook a randomized controlled trial of the efficacy of PQ in combination with CQ or AL at two sites in Oromia Region, Ethiopia.

## Methods

### Ethics

Ethical approval for the study was granted by the National Research Ethics Review Committee in Ethiopia (3.10/801/05), the Human Research Ethics Committee of the Northern Territory Department of Health and Menzies School of Health Research in Australia (HREC 2013–1938), the US Centers for Disease Control and Prevention Institutional Review Board B (6338.0), and the Columbia University Institutional Review Board (AAAK4706).

### Study design and participants

The study was designed as an open-label randomized controlled trial with four arms, conducted at the Bishoftu Malaria Control Center and the Batu Health Center in Oromia Region, in central Ethiopia (S1 Text). Bishoftu is approximately 50 km south of Addis Ababa, and Batu a further 115 km south. The main transmission season is between September and November, and healthcare facility data confirmed *P*. *vivax* to be the dominant malaria species. *Anopheles arabiensis* is the primary malaria vector, and the prevalence of malaria in Oromia Region was 0.4% during the 2011 national malaria indicator survey [10]. Patients seeking care with suspected malaria were screened for the following inclusion criteria: slide-confirmed mono-infection with *P*. *vivax*, age > 1 y, weight ≥ 5.0 kg, living within 20 km of the enrolling health facility, and axillary temperature ≥ 37.5°C or history of fever during the previous 48 h. Patients were excluded if they were pregnant or breastfeeding, had danger signs or severe manifestations of malaria, signs of severe malnutrition, slide-confirmed infection with any other *Plasmodium* species besides *P*. *vivax*, acute anaemia (haemoglobin [Hb] < 80 g/l) or history of haemolysis, known hypersensitivity to any of the study drugs, other significant comorbidities, or regular medication that could interfere with their antimalarial treatment. All participants or their guardian/caregiver agreed to finger prick sampling and provided written informed consent.

### Randomization

Patients were randomized in a two-stage process: (i) at enrolment, randomization to either CQ or AL schizonticidal treatment and (ii) on day 2, after G6PD testing, randomization into the PQ or no-PQ arms. Patients with severe or intermediate G6PD deficiency were excluded from the second randomization to PQ. The randomization sequence was computer-generated by one of the investigators and kept in sealed opaque envelopes. Randomization was done in blocks of eight for each site separately. Clinicians enrolled the patients, and nurses assigned the patients sequentially according to the sealed envelopes.

### Study procedures

#### Treatment

CQ (Micro Labs Limited, Tamil Nadu, India) was prescribed according to national guidelines at 25 mg base/kg over 3 d (10 mg base/kg on days 0 and 1, and 5 mg base/kg on day 2). AL (20 mg of artemether and 120 mg of lumefantrine; Novartis Pharmaceuticals Corporation, New York, NY, US) was administered twice daily for 3 d according to the manufacturer’s recommendations. The CQ and AL tablets used in the study were certified and provided by the World Health Organization Global Malaria Programme. Initial schizonticidal treatment with CQ was fully supervised, whereas for AL only the morning dose was supervised, and the second dose taken unsupervised at home. PQ (Sanofi-Aventis, Bridgewater, NJ, US) was prescribed at 0.25 mg/kg daily dose over 14 d starting on day 2, and administration was semi-supervised during the initial episode, with directly observed treatment (DOT) on days 2, 3, 7, 10, and 14; doses in between were self-administered. For all subsequent episodes of malaria, schizonticidal as well as PQ treatment was unsupervised.

All patients were observed for 60 min after treatment for adverse reactions or vomiting when administered under supervision. Patients vomiting their medication within the first 30 min received a repeat full dose; patients vomiting within 30–60 min received half the original dose. Patients failing treatment in any arm within 14 d were treated with quinine 10 mg/kg every 8 h for 7 d as per the national guideline.

#### Clinical procedures

During screening, a G6PD test was carried out on all patients, and a pregnancy test on all adult females. At enrolment, patients had a medical examination, a questionnaire was completed, and a capillary blood sample was collected for blood film examination and Hb measurement. Three 50-μl capillary blood spots were stored on filter papers (Whatman 903 and Whatman 3; GE Healthcare Biosciences, Westborough, MA, US). In consenting patients, venous blood samples were collected at enrolment and, if malaria recurred, on the day of recurrence.

Patients were asked to return for routine assessment daily for the first 3 d, then weekly until day 42, and thereafter monthly for an additional 10 mo. Patients were also asked to return to the clinic if they had signs or symptoms consistent with malaria, and those with recurrent clinical *P*. *vivax* malaria occurring more than 14 d following treatment received the same treatment as that allocated at enrolment, with subsequent follow-up according to the initial schedule.

A follow-up medical exam and blood film examination was undertaken on days 1, 2, 3, 7, 14, 21, 28, 35, and 42, and monthly thereafter for 10 mo. Adverse drug reactions and concomitant medications were recorded at every visit, with repeat Hb concentration measured on days 3, 7, 14, 21, 28, 35, and 42.

#### Laboratory procedures

G6PD status was determined by NADPH fluorescent spot test (Trinity Biotech, Bray, Ireland). Hb concentration was measured using a HemoCue Hb 301 instrument (HemoCue, Angelholm, Sweden). Blood film examination was undertaken by two certified microscopists, and all slides read independently. Blood smears with discordant results (differences between the two microscopists in species diagnosis, in parasite density of >50%, or in regard to the positivity of the smear) were re-examined by a third, independent microscopist, and parasite density was calculated by averaging the two closest counts.

PCR-based species confirmation was undertaken on all clinical samples on day 0 and the day of any recurrence until day 42 using a genus/*P*. *falciparum* duplex PET-PCR assay [11] and an in-house *P*. *vivax* PET-PCR assay. Parasite genotyping was undertaken using seven microsatellite markers as described previously [12,13] (S2 Text).

When recurrence of *P*. *vivax* occurred before day 28, whole blood concentrations of CQ and its metabolite desethylchloroquine (DCQ) were measured from dried blood spots collected on the day of recurrence using an HPLC method [14]. The limit of detection for both CQ and DCQ was 5 ng/ml. Levels < 5 ng/ml were designated as below detection.

### Outcome

The primary endpoint was the cumulative risk of *P*. *vivax* recurrence at day 28 and day 42 following treatment of the first episode of malaria, comparing AL with AL+PQ and CQ with CQ+PQ. A secondary endpoint was the cumulative risk at day 28 for AL compared with CQ and for AL+PQ compared with CQ+PQ. Other secondary endpoints included parasite and fever clearance, and cumulative risk and incidence rate of recurrences at the end of the study. Safety endpoints were the proportion of patients with adverse events (AEs) and serious AEs, the fractional change in Hb, the proportion of patients with a >25% drop in Hb between baseline and day 7, and the proportion of patients with anaemia (Hb < 100 g/l) on days 3 and 7. Haematological recovery at day 28 was defined as an Hb value on day 28 above baseline.

### Statistical analysis

The sample size was based on a power calculation assuming an efficacy of 68% at day 42 after CQ monotherapy and that the addition of PQ would increase this to 87%. A total of 97 in each group would achieve 90% power to detect this difference between groups. Adjusting the alpha level to 0.025 for multiple comparisons and estimating 15% loss to follow-up increased the proposed sample size to 120 per treatment arm.

Data were double-entered into a Microsoft Access database, and analyses conducted using STATA 14 (StataCorp, College Station, TX, US), according to an a priori statistical analysis plan (S3 Text). In view of the potential interaction between PQ and CQ [15] and between PQ and AL [16], comparisons between each of the arms were undertaken separately, rather than using a 2 × 2 factorial design approach, which assumes no interaction between the interventions. The cumulative risks were calculated by survival analyses (Kaplan—Meier) at day 28, day 42, and the end of the follow-up, and treatment groups compared using a Cox regression model. The proportional hazards assumption was assessed by visually comparing the log(cumulative hazard) by time of follow-up curves for each co-variable category and subsequently by fitting and comparing models with and without time of follow-up interaction terms. Genotyping results were used to calculate adjusted cumulative risk of recurrence by days 28 and 42 by censoring for heterologous infections, which may represent reinfection or relapse; for this analysis, only PCR-confirmed *P*. *vivax* infections at enrolment were included (S2 Text) [17]. The risks of recurrence after primary and secondary treatments were compared at 6 mo to ensure sufficient patient follow-up time. Incidence rates were calculated from microscopy results by dividing the number of *P*. *vivax* episodes by the number of person-years of observation. Incidence rate ratios between treatment arms were derived from a negative binomial regression model. The primary analysis was by intention to treat (ITT), and in the secondary analysis a modified ITT approach was used, in which patients receiving incorrect treatment or in whom no treatment information was available were censored on the day of the recurrence. Patients with intermediate or deficient G6PD status were not included in the second stage of randomization; they were included only in descriptive results and were excluded from any relevant comparisons between arms.

## Results

### Baseline characteristics

**Fig 1:**
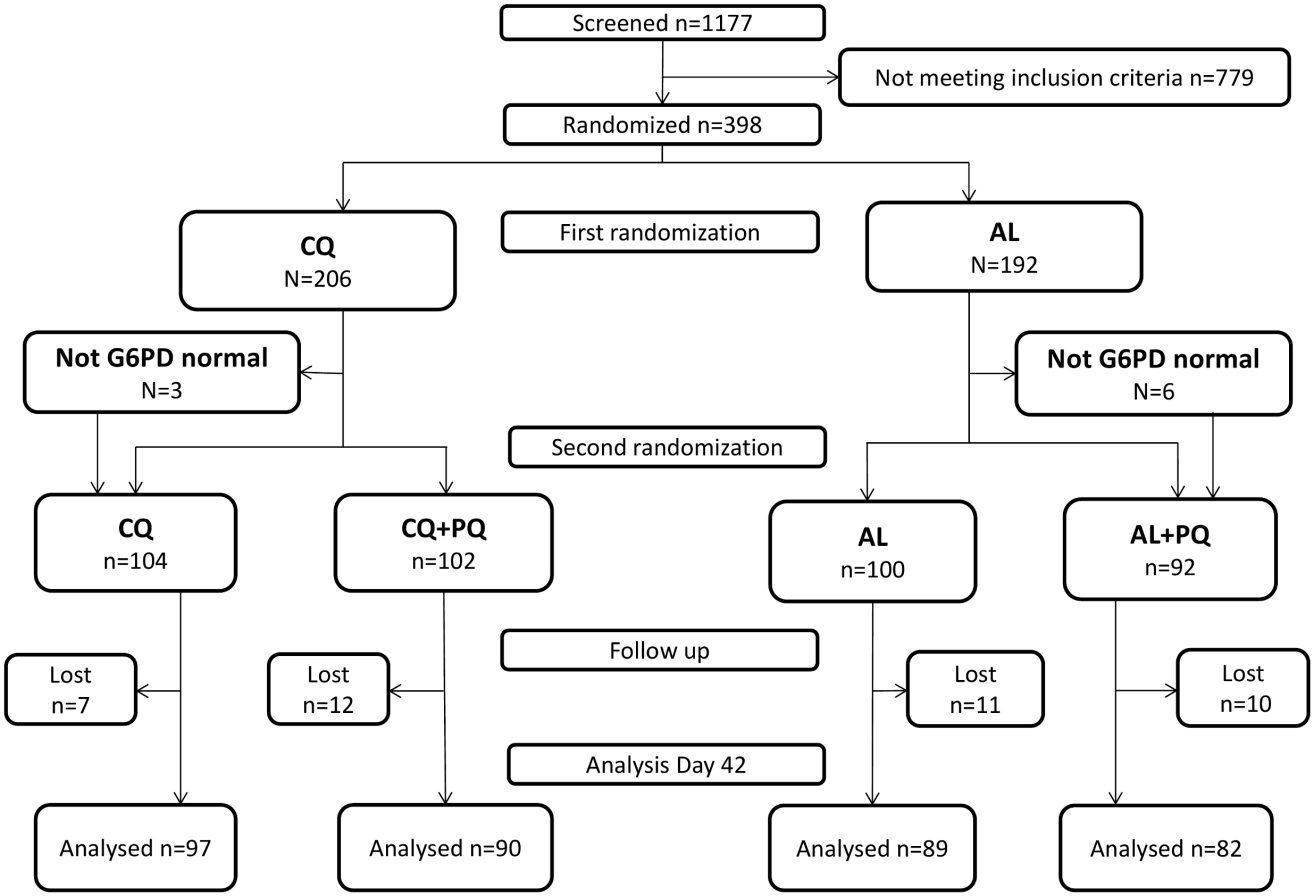
CONSORT flowchart of patient allocation for the day 42 outcome. AL, artemether-lumefantrine; AL+PQ, artemether-lumefantrine + primaquine; CQ, chloroquine; CQ+PQ, chloroquine + primaquine; G6PD, glucose-6-phosphate dehydrogenase; Lost, lost to follow-up.

The study was conducted between 8 November 2012 and 31 December 2014, during which a total of 1,177 patients were screened, and 398 (33.8%) enrolled in the study (Fig 1). In view of logistical constraints and the lack of further patients with malaria, the study was terminated at the end of the malaria season in 2014, after 82.9% (398/480) of the target sample size had been recruited.

The majority of patients (77.1%, 307/398) were enrolled at the Bishoftu Malaria Control Center. There were no important differences in baseline characteristics between the four treatment arms (Table 1) or in the proportion of patients lost to follow-up by day 42. G6PD deficiency was measured for 393 (98.7%) patients; missing values are mainly due to loss to follow-up before day 2. Nine (2.3%, 9/393) patients had intermediate G6PD results; they were randomized on day 0 for schizonticidal treatment, but did not receive PQ treatment (Table 1). In total, 98.2% (391/398) of patients completed a full course of schizonticidal treatment, of whom six (1.5%) vomited their dose within 1 h of administration (four in the CQ arm and two in the AL arm). One patient vomited doses of AL on day 1 and 2.

**Table 1:** Baseline characteristics of the study population.

Characteristic	Chloroquine (*n* = 104)	Artemether-lumefantrine (*n* = 100)	Chloroquine plus primaquine (*n* = 102)	Artemether-lumefantrine plus primaquine (*n* = 92)
**Health centre, *n* (percent)**				
Bishoftu	79 (76.0%)	73 (73.0%)	79 (77.5%)	76 (82.6%)
Batu	25 (24.0%)	27 (27.0%)	23 (22.5%)	16 (17.4%)
**Male, *n* (percent)**	64 (61.5%)	70 (70.0%)	68 (66.7%)	51 (55.4%)
**Age (years), median (IQR)**	18 (IQR 10.5–19.0)	18 (IQR 10.0–26.0)	17 (IQR 9.0–25.0)	18 (IQR 9.0–27.0)
**Age, *n* (percent)**				
≤5 y	7 (6.7%)	12 (12.0%)	10 (9.8%)	8 (8.7%)
5–15 y	31 (29.8%)	31 (31.0%)	37 (36.3%)	28 (30.4%)
>15 y	66 (63.5%)	57 (57.0%)	55 (53.9%)	56 (60.9%)
**Weight (kg), median (IQR)**	50.0 (29.0–56.0)	48.0 (24.5–56.5)	46.0 (22.0–54.0)	45.5 (22.0–57.0)
**Temperature (°C), median (IQR)**	36.8 (36.1–38.4)	37.3 (36.4–39.0)	37.3 (36.2–38.8)	37.0 (36.4–38.2)
**Fever, *n* (percent)**	37 (35.6%)	46 (46.0%)	43 (42.1%)	50 (54.3%)
**History of fever, *n* (percent)**	100 (98.0%)	98 (99.0%)	100 (99.0%)	90 (97.8%)
**Haemoglobin (g/l), mean (SD)**	136 (±17)	137 (±20)	134 (±19)	135 (±18)
**Parasitaemia (μl**^**−1**^), **geometric mean (95% CI)**	6,378 (5,133.1–7,924.3)	6,691 (5,464.7–8,193.0)	5,598 (4,492.6–6,975.4)	5,132 (4,036.8–6,523.6)
**Gametocyte carriage, *n* (percent)**	42 (40.4%)	47 (47.0%)	39 (38.2%)	43 (46.7%)
**G6PD status, *n* (percent)**				
Normal	100 (97.1%)	90 (93.8%)	102 (100.0%)	92 (100.0%)
Intermediate	3 (2.9%)	6 (6.3%)	0	0
Deficient	0	0	0	0
**Bednet use and insecticide use, *n* (percent)**				
Household ownership of a bednet	33 (34.7%)	42 (42.0%)	36 (35.3%)	37 (40.7%)
Patient slept under bednet on the night before enrolment	14 (13.5%)	17 (17.0%)	22 (21.6%)	22 (23.9%)
House was sprayed with insecticides	23 (22.1%)	22 (22.0%)	19 (18.6%)	18 (19.6%)

IQR, interquartile range; SD, standard deviation.

### Day 28 and 42 outcomes

**Fig 2:**
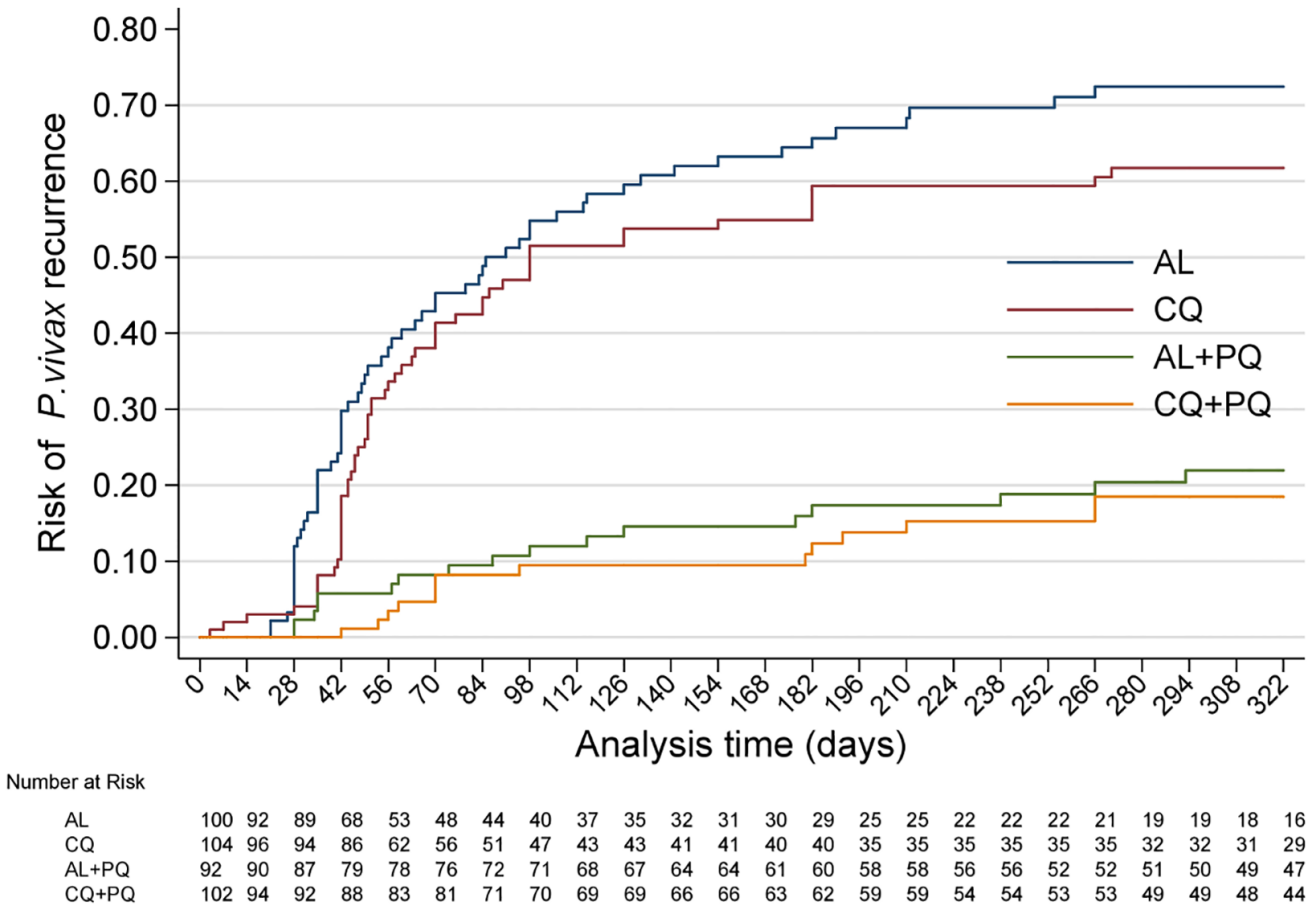
Cumulative risk of *P*. *vivax* recurrence in all four treatment arms over the entire follow-up time. AL, artemether-lumefantrine; AL+PQ, artemether-lumefantrine + primaquine; CQ, chloroquine; CQ+PQ, chloroquine + primaquine.

There were 17 recurrent *P*. *vivax* infections documented within 28 d of follow-up, and a further 34 between day 28 and 42 (27 in the AL arm, 18 in the CQ arm, five in the AL+PQ arm, and one in the CQ+PQ arm) (Table 2). An additional five patients presented with a *P*. *falciparum* infection before day 28, and one more between day 28 and 42. Patients in treatment arms that included PQ had significantly fewer recurrent malaria episodes than patients on schizonticidal therapy alone. By day 28, the cumulative risk for *P*. *vivax* recurrence was 4.0% (95% CI 1.5%–10.4%) for patients treated with CQ alone compared to 0% (95% CI 0%–4.0%) for those treated with CQ+PQ (*p <* 0.001). The corresponding risks were 12.0% (95% CI 6.8%–20.6%) following AL alone and 2.3% (95% CI 0.6%–9.0%) following AL+PQ (hazard ratio [HR] = 5.1 [95% CI 1.1–23.5], *p* = 0.034) (Fig 2; Tables 2 and 3).

**Table 2:** Treatment failure and risk and incidence of recurrent parasitaemia.

Time point	Outcome measure	CQ (*n* = 104)	CQ+PQ (*n* = 102)	AL (*n* = 100)	AL+PQ (*n* = 92)
**Day 28**	Lost to follow-up	6	8	8	6
	Early treatment failure	1	0	0	0
	Late treatment failure	3	0	11	2
	*P*. *falciparum* parasitaemia	2	3	0	0
	Adequate clinical and parasitological response	92	91	81	84
	Cumulative risk of *P*. *vivax*^a^	4.0 (1.5–10.4)	0 (0–4.0)	12.0 (6.8–20.6)	2.3 (0.6–9.0)
**Day 42**	Lost to follow-up	7	12	11	10
	Early treatment failure	1	0	0	0
	Late treatment failure	17	1	27	5
	*P*. *falciparum* parasitaemia	2	4	0	0
	Adequate clinical and parasitological response	77	85	62	77
	Cumulative risk of *P*. *vivax*^a^	18.7 (12.2–28.0)	1.2 (0.2–8.0)	29.9 (21.6–40.5)	5.9 (2.4–13.5)
**End of follow-up**	Lost to follow-up	15	39	22	28
	Early treatment failure	1	0	0	0
	Late treatment failure	56	14	62	17
	*P*. *falciparum* parasitaemia	3	5	0	0
	Adequate clinical and parasitological response	29	44	16	47
	Cumulative risk of *P*. *vivax*^a^	61.7 (51.9–71.7)	20.5 (13–31.5)	72.4 (62.5–81.6)	22.0 (14.2–33.1)
	Incidence of *P*. *vivax* recurrence^b^	2.2 (1.8–2.6)	0.4 (0.3–0.6)	2.3 (1.9–2.7)	0.5 (0.3–0.7)
	Adjusted incidence of *P*. *vivax* recurrence^c^	2.3 (1.9–2.7)	—	2.2 (1.8–2.6)	—
	Incidence of any *Plasmodium* recurrence^b^	2.2 (1.9–2.7)	0.5 (0.4–0.7)	2.3 (1.9–2.7)	0.5 (0.4–0.7)
	Adjusted incidence of any *Plasmodium* recurrence^c^	2.3 (1.9–2.8)	—	2.2 (1.9–2.7)	—

^a^Cumulative risk derived from survival analysis, percent (95% CI).

^b^Episodes per person-year of observation (95% CI); calculated from the duration of follow-up from first to last visit. Patients receiving antimalarial treatment were assumed to have a period of 28 d of post-treatment prophylaxis after CQ treatment and 14 d of post-treatment prophylaxis after AL treatment, and this period was subtracted from their total period of observation. This analysis included patients not randomized to PQ or no PQ.

^c^Excluding patients not randomized to PQ or no PQ.

AL, artemether-lumefantrine; AL+PQ, artemether-lumefantrine + primaquine; CQ, chloroquine; CQ+PQ, chloroquine + primaquine.

**Table 3:** Hazard ratios and incidence rate ratios.

Time point	Measure	CQ versus CQ+PQ^a^	AL versus AL+PQ^a^	AL versus CQ	AL+PQ versus CQ+PQ
**Day 28**	Hazard ratio	—, *p* = 0.0001	5.1 (1.1–23.5), *p* = 0.034	2.9 (0.9–9.0), *p* = 0.07	—, *p* = 0.08
**Day 42**	Hazard ratio	18.5 (2.5–138.5), *p* = 0.005	5.9 (2.3–15.3), *p <* 0.001	1.8 (1.0–3.2), *p* = 0.059	5.4 (0.6–46.6), *p* = 0.122
**End of follow-up**	Hazard ratio	5.4 (3.0–9.7), *p <* 0.001	5.2 (3.0–9.0), *p <* 0.001	1.3 (0.9–1.9), *p* = 0.127	1.3 (0.6–2.6), *p* = 0.523
	Incidence rate ratio	5.1 (2.9–9.1), *p <* 0.001	6.4 (3.6–11.3), *p <* 0.001	0.9 (0.6–1.4), *p* = 0.530	1.1 (0.5–1.9), *p* = 0.964

Hazard ratios (95% CIs) calculated from Cox regression. Incidence rate ratios (95% CIs) calculated at the end of follow-up.

^a^Excluding patients not randomized to PQ versus no PQ.

AL, artemether-lumefantrine; AL+PQ, artemether-lumefantrine + primaquine; CQ, chloroquine; CQ+PQ, chloroquine + primaquine.

By day 42, the risk of recurrence had risen to 18.7% (95% CI 12.2%–28.0%) in the CQ arm and 1.2% (95% CI 0.2%–8.0%) in the CQ+PQ arm (HR = 18.5 [95% CI 2.5–138.5], *p* = 0.005). The corresponding risk for patients in the AL arm was 29.9% (95% CI 21.6%–40.5%) compared to 5.9% (95% CI 2.4%–13.5%) in the AL+PQ arm (HR = 5.9 [95% CI 2.3–15.3], *p <* 0.001) (Fig 2; Tables 2 and 3). In those not prescribed PQ, the risk of recurrence by day 42 was greater following AL than CQ, although this difference was of borderline significance (HR = 1.8 [95% CI 1.0–3.2], *p* = 0.059) (Table 3).

Genotyping was feasible in 90.9% (362/398) of parasite isolates on day 0 and in 90.2% (46/51) of paired isolates from patients with recurrence prior to day 42. In total, 47.8% (22/46) of the paired *P*. *vivax* isolates that could be assessed were homologous (S2 Text).

Blood chloroquine concentrations on the day of recurrence could be measured in two of the four patients with recurrent infection before day 28 in the CQ arm, both of whom had CQ blood concentrations greater than 100 nM.

### Parasite and fever clearance

By day 1, 60.2% (124/206) of those treated with CQ were still parasitaemic compared to 31.8% (61/192) of those treated with AL regimens (odds ratio = 3.3 [95% CI 2.2–5.0], *p <* 0.001). By day 2, the prevalence of parasitaemia was 6.8% in the CQ arms and 2.6% in the AL arms. By day 3, only two patients in the CQ arms remained parasitaemic (Table 4).

**Table 4:** Parasite and fever clearance by treatment arm.

Time point	Chloroquine	Artemether-lumefantrine	Chloroquine plus primaquine	Artemether-lumefantrine plus primaquine
**Parasitaemia**				
Day 1	64.4% (67/104)	35.0% (35/100)	55.9% (57/102)	28.3% (26/92)
Day 2	4.8% (5/104)	5.0% (5/100)	8.8% (9/102)	0% (0/92)
Day 3	1.9% (2/104)	0% (0/100)	0% (0/102)	0% (0/92)
**Fever**				
Day 1	10.5% (4/38)	0% (0/46)	2.4% (1/42)	2.6% (1/39)
Day 2	0% (0/37)	0% (0/45)	0% (0/43)	2.6% (1/39)
Day 3	0% (0/36)	0% (0/45)	0% (0/42)	2.6% (1/38)

Of the 166 patients with documented fever at enrolment, 96.4% (159/165) were afebrile within 24 h, with 98.8% (84/85) in the AL arms compared to 93.8% (75/80) in the CQ arms (*p* = 0.109) (Table 4).

### Antirelapse efficacy

After 1 y of follow-up, 150 patients had experienced at least one recurrent episode of *P*. *vivax* determined by microscopy (57 after CQ, 62 after AL, 14 after CQ+PQ, and 17 after AL+PQ), and a further eight had had *P*. *falciparum* infections (three following CQ and five after CQ+PQ) (Table 2). The risk of any recurrence of *P*. *vivax* was 61.7% (95% CI 51.9%–71.7%) following CQ alone compared to 72.4% (95% CI 62.5%–81.6%) following AL alone (*p* = 0.127). Compared to CQ or AL alone, the risk of recurrence was significantly lower when treatment was combined with PQ: 20.5% (95% CI 13.0%–31.5%) following CQ+PQ (HR = 5.4 [95% CI 3.0–9.7] compared to CQ alone, *p <* 0.001) and 22.0% (95% CI 14.2%–33.1%) following AL+PQ (HR = 5.2 [95% CI 3.0–9.0] compared to AL alone, *p <* 0.001). There was no difference in the risk of recurrence at the end of the study between patients treated with CQ+PQ and AL+PQ (Fig 2; Tables 2 and 3).

Recurrent *P*. *vivax* infections occurred later in the PQ arms than the monotherapy arms. The median time of the first recurrence was 82.5 d (interquartile range [IQR] 61.0–186.5) for CQ+PQ compared to 51 d (IQR 42.0–86.0) for CQ alone (*p* = 0.006) and 87 d (IQR 35.0–117.0) for AL+PQ compared to 49.5 d (IQR 35.0–98.0) for AL alone (*p* = 0.040). In the no-PQ arms, 90.8% (108/119) of the recurrent parasitaemias occurred within the first 6 mo.

**Fig 3:**
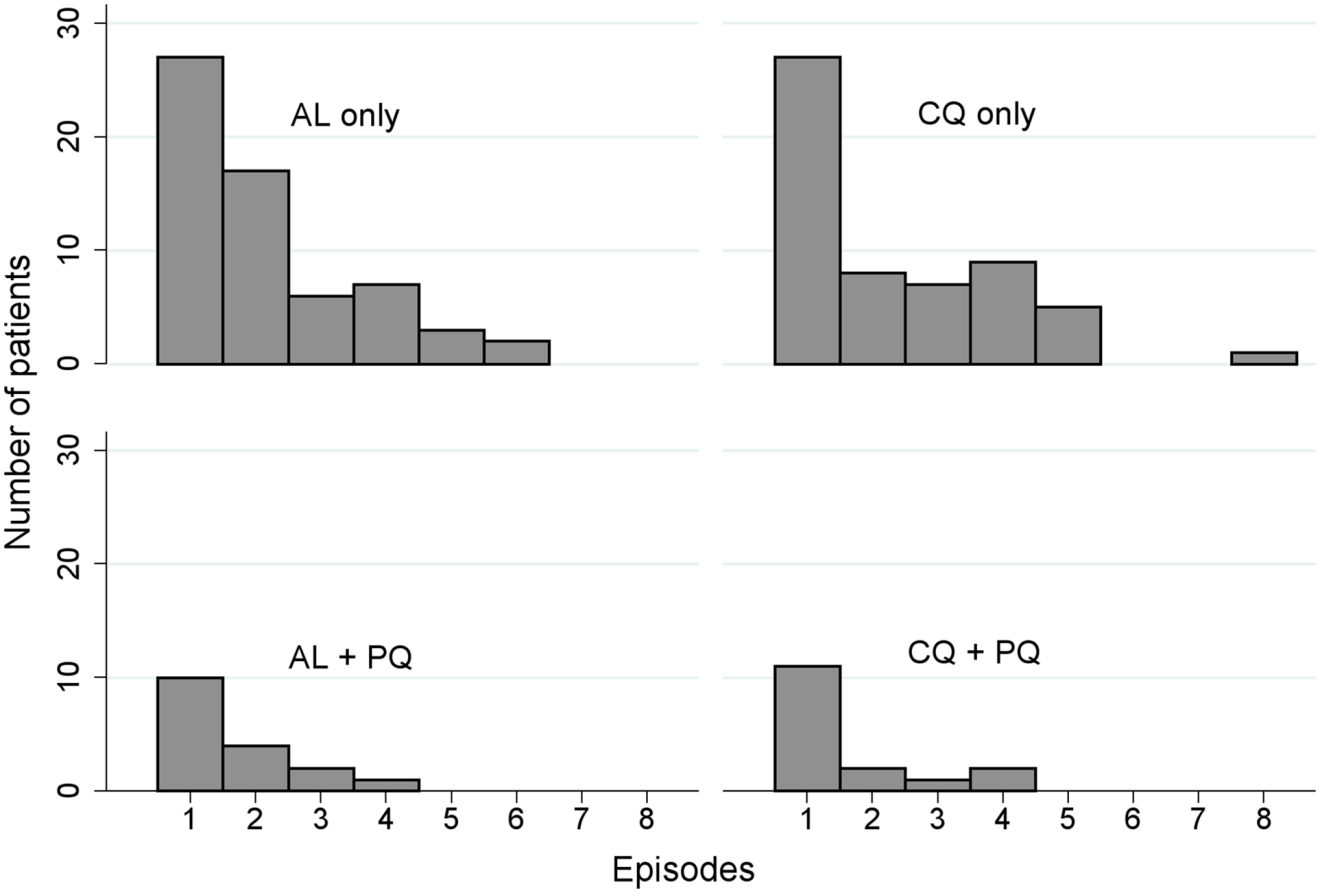
Histogram of the distribution of the number of recurrent *P*. *vivax* infections occurring during the 1-y follow-up period. AL, artemether-lumefantrine; AL+PQ, artemether-lumefantrine + primaquine; CQ, chloroquine; CQ+PQ, chloroquine + primaquine.

A total of 322 *P*. *vivax* recurrent infections were detected by microscopy during follow-up (134 after AL, 133 after CQ, 29 after AL+PQ, and 26 after CQ+PQ) (Fig 3). The incidence rate was 2.3 (95% CI 1.9–2.7) episodes per person-year of observation (PYO) for AL, 2.2 (95% CI 1.8–2.6) for CQ, 0.5 (95% CI 0.3–0.7) for AL+PQ, and 0.4 (95% CI 0.3–0.6) for CQ+PQ (Table 3).

### Partial primaquine dose and unsupervised primaquine treatment

The initial treatment with PQ was supervised on scheduled clinic days, and adherence assessed by tablet review at the subsequent visit. The total PQ dose administered per unit body weight was 3.4 mg/kg (IQR 2.8–4.0) in the AL+PQ arm and 3.5 mg/kg (IQR 2.6–4.0) in the CQ+PQ arm. The histogram of PQ dose distribution shows distinct populations that correspond to patients who received a total PQ dose below 2.6 mg/kg and the rest (S1 Fig). There were 27 patients in the CQ+PQ arm and 22 patients in the AL+PQ arm who received a dose below 2.6 mg/kg, and these patients appeared to be at greater risk of recurrence by 6 mo compared to those who received a dose >2.6 mg/kg: HR = 3.8 (95% CI 1.1–13.0) for CQ+PQ and HR = 2.8 (95% CI 1.0–8.0) for AL+PQ, *p* = 0.036 and *p* = 0.059, respectively.

For all subsequent recurrences, only the first dose of the antimalarial drugs was supervised. The risk of recurrence after 6 mo of follow-up between the primary and secondary treatments did not differ significantly for patients in either the CQ arm (HR = 1.2 [95% CI 0.8–1.84], *p* = 0.5) or the AL arm (HR = 0.8 [95% CI 0.5–1.3], *p* = 0.4). However, patients were at significantly greater risk of recurrent *P*. *vivax* after the unsupervised PQ retreatment than after the partially supervised initial PQ treatment: 86.8% (95% CI 77.3%–92.5%) versus 63.2% (95% CI 31.7%–93.3%) in patients in the CQ+PQ arm (HR = 3.9 [95% CI 1.3–11.4], *p* = 0.014) and 84.0% (95% CI 74.0%–90.4%) versus 51.9% (95% CI 24.4%–73.7%) in patients in the AL+PQ arm (HR = 3.5 [95% CI 1.4–8.9], *p* = 0.008) (S2 Fig).

### Safety

A total of 358 AEs were reported, but no serious AEs. There was no difference in the occurrence of any of the categories of AEs between treatment arms or by total PQ dosage received. The most frequent AEs were headache (25.5%), fever (18.8%), chills (9.2%), cough (7.0%), vomiting (6.7%), body pain (5.0%), abdominal pain (4.8%), weakness (4.2%) and diarrhoea (4.0%) (Table 5).

**Table 5:** Adverse events by treatment arm.

Adverse events (*n* = 358)	Total	Chloroquine	Artemether-lumefantrine	Chloroquine plus primaquine	Artemether-lumefantrine plus primaquine	*p*-Value
**Headache**	91 (25.5%)	27 (29.7%)	29 (31.9%)	19 (20.9%)	16 (17.6%)	0.68
**Fever**	67 (18.8%)	13 (19.4%)	18 (26.9%)	16 (23.9%)	20 (29.9%)	0.16
**Chills**	33 (9.2%)	11 (33.3%)	12 (36.4%)	5 (15.2%)	5 (15.2%)	0.43
**Cough**	25 (7.0%)	8 (32.0%)	5 (20.0%)	8 (32.0%)	4 (16.0%)	0.53
**Vomiting**	24 (6.7%)	7 (29.2%)	5 (20.8%)	7 (29.2%)	5 (20.8%)	0.79
**Body pain**	18 (5.0%)	3 (16.7%)	6 (3.3%)	5 (27.8%)	4 (22.2%)	0.77
**Abdominal pain**	17 (4.8%)	7 (41.2%)	3 (17.6%)	4 (23.5%)	3 (17.6%)	0.55
**Weakness**	15 (4.2%)	5 (33.3%)	5 (33.3%)	4 (26.7%)	1 (6.7%)	0.59
**Diarrhoea**	15 (4.0%)	4 (26.7%)	3 (20.0%)	4 (26.7%)	4 (26.7%)	0.86
**Fever and headache**	13 (3.6%)	1 (7.7%)	5 (38.5%)	2 (15.4%)	5 (38.5%)	0.20
**Nausea**	12 (3.4%)	4 (33.3%)	5 (41.7%)	1 (8.3%)	2 (16.7%)	0.54

Data are given as *n* (percent).

The nadir of Hb concentration occurred on day 3 irrespective of treatment. The mean percentage fall from baseline by day 3 was 6.3% (95% CI 5.0%–7.7%) in the CQ arm, 5.7% (95% CI 4.3%–7.1%) in the AL arm, 5.9% (95% CI 4.4%–7.4%) in the CQ+PQ arm, and 7.6% (95% CI 5.1%–10.0%) in the AL+PQ arm (*p* = 0.577). On day 3, 3.6% (7/195) of patients treated with a PQ regimen had anaemia (Hb < 100 g/l) compared to 4.2% (8/191) of those treated with a no-PQ regimen (*p* = 0.08). By day 28, haematological recovery occurred in 211 (70.8%) patients. Hb concentration did not fall below 80 g/l in any of the patients during the study period, and no patient required blood transfusion.

## Discussion

This study highlights that the combination of PQ with either CQ or AL reduced the risk of early recurrence (within 42 d) of *P*. *vivax* by up to 3-fold, and decreased the risk of recurrence over 1 y by 2- to 3-fold, compared to CQ or AL alone. PQ (3.5 mg/kg over 14 d) was well tolerated, without significant adverse effects. Recent clinical trials have highlighted the declining efficacy of CQ against *P*. *vivax* in Ethiopia [18–20], with the risk of recurrence at day 28 ranging from 14% [21] to 22% [20]. Our study provides evidence of low-grade CQ resistance [4,22], with 3.8% of patients having recurrence by day 28 in the presence of adequate CQ blood concentration (>100 nM). However, despite CQ’s compromised efficacy, the risk of recurrence was almost 2-fold greater in patients treated with AL alone compared to those treated with CQ alone, although this difference did not reach statistical significance (*p* = 0.059). In equatorial regions endemic for *P*. *vivax*, including Ethiopia, the risk of *P*. *vivax* relapse is generally high, with the first recurrence occurring about 21 d following the initial treatment [23]. Lumefantrine has an elimination half-life of 3–6 d, and, by 16 d, drug concentrations have fallen well below the minimum inhibitory concentration for the parasite and thus are no longer sufficient to prevent relapsing infections. Conversely, the slower elimination of CQ affords prolonged post-treatment prophylaxis, capable of suppressing early relapses [24]. By day 42, both CQ and lumefantrine levels are expected to be below the minimum inhibitory concentration, and, hence, in the absence of PQ, it is not surprising that there was no difference in the incidence of recurrent infections beyond that time.

The prolonged duration of the study and repeated administration of the same treatment regimen for each episode of malaria allowed quantification of the cumulative risk of *P*. *vivax* and incidence of recurrence over a 12-mo period. The incidence of infection reflects the likely impact that would result from corresponding policy change. Patients receiving CQ or AL alone had four or five recurrences, with an overall incidence rate of approximately two episodes per PYO. The addition of a supervised 14-d regimen of PQ resulted in a 4-fold reduction in the incidence rate, from 2 to 0.5 episodes per PYO. Radical cure with PQ is a function of the total mg/kg dose administered [25,26], and this was highlighted by a clear dose response in our study. Patients who received a total PQ dose below 2.6 mg/kg had significantly more recurrences than those who received a greater dose: almost 4-fold more in the CQ+PQ arm and 3-fold more in the AL+PQ arm (*p <* 0.05).

There was also a 3- to 4-fold greater risk of recurrence following treatment of patients’ first recurrent parasitaemia (which was unsupervised) compared to that following treatment of their initial parasitaemia (which was semi-supervised). Although this non-randomized comparison is vulnerable to bias, there was no difference in the risk of recurrence following the first and second treatments with CQ or AL monotherapy, suggesting that the difference was likely due to poor adherence to an unsupervised 14-d regimen of PQ. These findings are consistent with previous studies that have shown PQ adherence falling to less than 30% when unsupervised [27–29]. Significantly lower rates of recurrent parasitaemia were seen in a Thai study when PQ administration was directly observed compared to when it was unsupervised [30], although a difference was not observed in a study in Pakistan [31]. Our study emphasises the need for improved delivery of radical cure, using DOT, shorter treatment courses, and greater emphasis on patient and community education to promote the importance of radical cure and the need to continue treatment after resolution of symptoms [32].

There is reluctance of some healthcare providers to prescribe PQ, mainly due to concerns over its safety and potential for causing severe haemolysis [33]. To ensure safe delivery of PQ, WHO recommends prior testing for G6PD deficiency [34]. However, not many *P*. *vivax* endemic countries have implemented this yet. Further work to understand the barriers to routine G6PD testing is currently underway. In our study, PQ was administered to individuals confirmed to be G6PD normal at a total dose of 3.5 mg/kg (a low-dose radical cure regimen) [26]. This regimen was well tolerated, with no significant difference in the number or type of AEs between the PQ and no-PQ study arms. The predominant decline in Hb concentration occurred between enrolment and day 3, although PQ treatment only commenced on day 2. Hb recovery began after day 3; the greatest fall in Hb was thus a consequence of malaria rather than being drug-induced. Furthermore, treatment with PQ resulted in a significant reduction in *P*. *vivax* recurrences, each of which were associated with repeated bouts of haemolysis and a cumulative risk of anaemia [5,35].

There were a number of limitations of the study. First, the omission of routine Hb measurements after day 42, except in patients with recurrent malaria, prevented quantification of the long-term effects of recurrent episodes of malaria on the cumulative risk of anaemia. Further studies are needed to define the risk and benefits of alternative PQ dosing strategies in different endemic settings. Second, whilst all of the early recurrent parasitaemias were genotyped and compared with the pre-treatment parasitaemia, this comparison was not undertaken for recurrent infections after day 42. Heterologous recurrences occurring within the first 42 d can be due to either reinfection or relapse events but not true recrudescences. Parasite genotyping allows these events to be censored, enabling a better estimate of early efficacy. However, genotyping of late recurrences cannot distinguish between relapsing infections, which can be either homologous or heterologous, and reinfections. For this reason, we used a conservative approach to quantify and compare the cumulative risk and incidence of all recurrent *P*. *vivax* infections irrespective of whether they were homologous or heterologous. Third, the early termination of our trial reduced the final sample size. However, loss to follow-up was lower than assumed in the prior power calculation, and the final sample size enrolled into the study still achieved a power of greater than 80% for the primary outcomes of the study.

In conclusion, although there was evidence of low-grade CQ resistance of *P*. *vivax* in our study, the risk of early recurrence appeared greater following AL, likely due to relapsing infections emerging after the post-treatment prophylaxis of AL had waned. When administered as monotherapy, there was little benefit of changing the first-line therapy for vivax malaria from CQ to AL in this region of Ethiopia. However, PQ treatment should be included in future revision of the national treatment guidelines to decrease relapse rates, with likely benefits in reducing both malarial anaemia and transmission potential. Better methods of ensuring adequate adherence will be needed if the public health impact is to be maximised.

## Supporting information

S1 Fig Histogram of primaquine dose distribution. (DOCX)

S2 Fig Cumulative risk of *P. vivax* parasitaemia after complete and incomplete primaquine treatment of primary infections and unsupervised primaquine treatment of recurrent infections. (DOCX)

S1 PRISMA Checklist Checklist according to PRISMA guidelines. (DOC)

S1 Text Study protocol. (PDF)

S2 Text Genotyping methods and results. (PDF)

S3 Text Statistical analysis plan. (PDF)

## Abbreviations

AE: adverse event
AL: artemether-lumefantrine
CQ: chloroquine
DOT: directly observed treatment
G6PD: glucose-6-phosphate dehydrogenase
Hb: haemoglobin
HR: hazard ratio
IQR: interquartile range
PQ: primaquine
PYO: person-year of observation
