# Correction: Comparison of artemether-lumefantrine and chloroquine with and without primaquine for the treatment of *Plasmodium vivax* infection in Ethiopia: A randomized controlled trial

**DOI:** 10.1371/journal.pmed.1002677

**Published:** 2018-10-04

**Authors:** Tesfay Abreha, Jimee Hwang, Kamala Thriemer, Yehualashet Tadesse, Samuel Girma, Zenebe Melaku, Ashenafi Assef, Moges Kassa, Mark D. Chatfield, Keren Z. Landman, Stella M. Chenet, Naomi W. Lucchi, Venkatachalam Udhayakumar, Zhiyong Zhou, Ya Ping Shi, S. Patrick Kachur, Daddi Jima, Amha Kebede, Hiwot Solomon, Addis Mekasha, Bereket Hailegiorgis Alemayehu, Joseph L. Malone, Gunewardena Dissanayake, Hiwot Teka, Sarah Auburn, Lorenz von Seidlein, Ric N. Price

The numbers in the second paragraph in the section entitled, “Partial primaquine dose and unsupervised primaquine treatment” are incorrect owing to a transcription error in the final preparation of the paper.

The paragraph should read: “For all subsequent recurrences, only the first dose of the antimalarial drugs was supervised. The risk of recurrence after 6 mo of follow-up between the primary and secondary treatments did not differ significantly for patients in either the CQ arm (HR = 1.2 [95% CI 0.8–1.84], p = 0.5) or the AL arm (HR = 0.8 [95% CI 0.5–1.3], p = 0.4). However, patients were at significantly greater risk of recurrent *P. vivax* after the unsupervised PQ retreatment than after the partially supervised initial PQ treatment: 36.8% (95%CI 16.6%–68.3%) versus 14.7% (95% CI 8.6%–24.6%) in patients in the CQ+PQ arm (HR = 3.9 [95% CI 1.3–11.4], p = 0.014) and 48.1% (95%CI 26.5%–75.6%) versus 17.5% (95%CI 10.7%–27.8%) in patients in the AL+PQ arm (HR = 3.5 [95% CI 1.4–8.9], p = 0.008) (S2 Fig).”
