# Supplementary material for: Comparison of artemether-lumefantrine and chloroquine with and without primaquine for the treatment of Plasmodium vivax infection in Ethiopia: A randomized controlled trial
Source: PLoS Med. 2017 May 16;14(5):e1002299. doi: 10.1371/journal.pmed.1002299 (PMC5433686; doi:10.1371/journal.pmed.1002299)
Supplement: S1 Fig — (DOCX) [file pmed.1002299.s001.docx]

**S1 Figure**

**S1 Figure: Histogram of PQ dose distribution**
