# Supplementary material for: Comparison of artemether-lumefantrine and chloroquine with and without primaquine for the treatment of Plasmodium vivax infection in Ethiopia: A randomized controlled trial
Source: PLoS Med. 2017 May 16;14(5):e1002299. doi: 10.1371/journal.pmed.1002299 (PMC5433686; doi:10.1371/journal.pmed.1002299)
Supplement: S2 Fig — (DOCX) [file pmed.1002299.s002.docx]

**S2 Figure**

**S2 Figure: Cumulative risk of *P. vivax* parasitaemia after complete and incomplete PQ treatment of primary and unsupervised PQ treatment of recurrent infections**

Footnote Treatment arms: A) Primary episode of P. vivax with complete PQ treatment (supervised on alternate days). B) Primary episode with incomplete PQ treatment (total dose of PQ below 2.6mg/kg) C) Recurrent episode treated with unsupervised PQ.
