# Supplementary material for: Comparison of artemether-lumefantrine and chloroquine with and without primaquine for the treatment of Plasmodium vivax infection in Ethiopia: A randomized controlled trial
Source: PLoS Med. 2017 May 16;14(5):e1002299. doi: 10.1371/journal.pmed.1002299 (PMC5433686; doi:10.1371/journal.pmed.1002299)
Supplement: S1 Text — (PDF) [file pmed.1002299.s004.pdf]

Ethiopia antimalarial *in vivo* efficacy study 2012: Evaluating the efficacy of artemether-lumefantrine alone compared to artemether-lumefantrine plus primaquine and chloroquine alone compared to chloroquine plus primaquine for *Plasmodium vivax* infection

COLLABORATORS

Centers for Disease Control and Prevention (CDC):
Jimee Hwang, MD MPH (ethics number 1008) will be responsible for protocol development, data management and analysis, and preparation of reports
S. Patrick Kachur, MD MPH (ethics number 6519) will supervise protocol development and preparation of reports.
Joseph L. Malone, MD (ethics number 7488) will review protocol development, supervise the field work, and preparation of reports.
Michael Green PhD (ethics number 17614) will conduct the drug level testing.
Venkatachalam Udhayakumar PhD (ethics number 13427) will provide oversight of molecular laboratory work at CDC.
Ya Ping Shi MD (ethics number 11249) will provide assistance with glucose-6-phosphate dehydrogenase deficiency testing and molecular testing.

Ethiopia Health and Nutrition Research Institute (EHNRI):
Daddi Jima, MD, MPH will review protocol development and preparation of reports.
Moges Kassa, MSc will be responsible for protocol development and provide oversight of molecular laboratory work at EHNRI.
Ashenafi Assefa, MSc will be responsible for protocol development and provide oversight of molecular laboratory work at EHNRI.

Federal Ministry of Health (FMOH):
Hiwot Solomon, will review protocol development and preparation of reports.

Columbia University ICAP:
Zenebe Melaku, MD will supervise protocol development and the implementation of the study.
Tesfay Abreha, MSc, MPH will assist in protocol development, be responsible for the implementation of the study, and the training and supervision of staff members.
Samuel Girma, MD will assist in the implementation of the study and the training and supervision of staff members.
Yehualashet Tadesse, MD, MSc will assist in the implementation of the study and the training and supervision of staff members.
Bereket Hailegiorgis Alemayehu, MD MSc will assist in protocol development and the implementation of the study, and the training and supervision of staff members.
David Hoos, MD MPH will supervise protocol development and the implementation of the study.

Oromia Regional Health Bureau:
Addis Mekasha, BSc, MPH will facilitate logistical support for the project at the regional level.

United States Agency for International Development (USAID):
Hiwot Teka, BSC MSc will review protocol development and preparation of reports.

University of Menzies:
Ric Price, PhD will assist in protocol development and preparation of reports and provide oversight during follow-up.
Lorenz von Seidlein, MD, PhD will assist in protocol development and preparation of reports and provide oversight during follow-up.

Expected Dates: September 2012- September 2013
Location: Oromia Regional State, Ethiopia
Version date: July 10, 2012

SUMMARY 4
INTRODUCTION 5
OBJECTIVES...6
STUDY DESIGN...6
Study Sites 6
Drugs...7
Population 9
SCREENING AND ENROLLMENT PROCEDURES 10
FOLLOW-UP PROCEDURES...12
END-POINTS...13
DATA HANDLING AND ANALYSIS 16
HANDLING OF UNEXPECTED OR ADVERSE EVENTS 17
ETHICAL ISSUES 18
DISSEMINATION, NOTIFICATION AND REPORTING OF RESULTS 21
REFERENCES...22
ANNEX...24
ANNEX I *P. vivax* treatment outcomes...24
ANNEX II Severe malaria definitions...25
ANNEX III Weight for Height Chart 26
ANNEX IV Drugs with antimalarial activity that should not be used during the study 27
ANNEX V Schedule of follow-up activities..28
ANNEX VI Study Schedule 30
ANNEX VII Consent/Assent Form 32
ANNEX VIII Material Transfer Agreement 40

SUMMARY

Following the rapid development of significant drug resistance of *Plasmodium falciparum* (Pf) to chloroquine and then sulfadoxine-pyrimethamine, artemether- lumefantrine (Coartem or AL) was adopted as first line therapy in Ethiopia in 2004. According to the current national malaria diagnosis and treatment guidelines updated in 2012 [1], first-line treatment for uncomplicated *P. falciparum* infection is AL. First-line treatment for *Plasmodium vivax* (Pv) is chloroquine (CQ) alone in malarious areas and with primaquine in non-malarious areas at health center and hospital level. WHO recommends treatment of Pv with CQ or an artemisinin-based combination therapy (ACT) in combination with primaquine [2]. For all clinical infection without laboratory confirmation, AL is the first-line treatment since AL is effective against both Pf and Pv. Thus, in Ethiopia, where treatment for malaria without laboratory confirmation occurs frequently, Pv is often treated with AL as the standard of care. Similarly, the recommended drug for mixed infection with Pf and Pv is AL. Now with wide-spread use of AL and CQ and with evidence that malaria laboratory testing is occurring in about half of those suspected with clinical evidence of malaria infection, we propose to conduct an antimalarial efficacy study to monitor the effectiveness of these therapies in Ethiopia and to determine how efficacious these drugs remain for Pv. In addition, with high rates of relapse with *P. vivax* infection, the efficacy and safety of co-administering primaquine will be assessed. This information will inform future policy changes with respect to appropriate antimalarial strategies.

In this proposal, patients aged above 1 year with symptomatic malaria presenting to health centers will be enrolled for treatment with AL, AL+PQ, CQ, or CQ+PQ, for vivax infection. Phase 1 of the study will monitor the clinical, parasitological, and hematological parameters for *P. vivax* infection over a 42-day follow-up period, which will be used to evaluate drug efficacy. Phase 2 will continue monthly follow-up of these patients for one year to assess frequency of recurring vivax infections. Results from this research study will be used to assist Ethiopia in assessing their current national malaria drug policies.

INTRODUCTION

Malaria in Ethiopia is highly unstable, with potential for epidemics. In Ethiopia, malaria transmission is largely determined by climate and altitude. Most of the transmission occurs between September and December, after the main rainy season from June to August. Malaria is the leading communicable disease in Ethiopia. In 2009–2010, malaria was the most common cause of outpatient visits and admissions, accounting for 14% of all visits and 9% of admissions [3]. About 75% of the country is malarious (defined as areas <2000 m), with about 68% (i.e. 52 million) of the country's total population living in areas at risk of malaria.

P. falciparum (Pf) and *P. vivax* (Pv) are the two dominant parasite species with relative frequencies of 60% and 40%, respectively, although this relative proportion varies both temporally and spatially, with published ranges of 22-89% for *P. falciparum* and 11-67% for *P. vivax* [4-6]. Chloroquine (CQ) resistant Pf was first reported from Africa in 1978 [7] and became a major public health threat to Ethiopia in the 1990s. With reports of wide-spread Pf resistance to CQ [8] and sulphadoxine-pyrimethamine (SP) [9, 10], the Federal Ministry of Health (FMOH) adopted artemether-lumefantrine (AL) for first-line treatment of uncomplicated *P. falciparum* malaria in 2004 [11]. Although CQ remained the first-line treatment for *P. vivax* malaria in Ethiopia, AL is used widely for all cases of clinical malaria where diagnostics to determine the specific malaria species are not available and for mixed infections.

Following the first report of CQ resistant *P. vivax* in 1989 from Papua New Guinea [12], decreased CQ susceptibility of <50% [13] has been reported from Indonesia [14-17] and Papua New Guinea [18]. Decreased susceptibilities of 50-90% have been reported from Burma/Myanmar [19], Vietnam [20, 21], Turkey [22, 23], Colombia [24], and Brazil [25]. Overall, CQ resistant *P. vivax* has remained rare in Africa. In 1996, Ethiopia published its first report of CQ resistance, with 2% (5/255) of study patients failing at day 7 [26]. Subsequent reports from Ethiopia documented presence of CQ-resistant *P. vivax*, but at levels not exceeding 5% [27-29]. A recent study from SNNPR noted 13% treatment failure but they did not measure CQ levels [30].

P. vivax unlike *P. falciparum* has a dormant stage [hypnozoites] that can persist in the liver and cause relapses by invading the bloodstream weeks, or even years later. Both CQ and AL treat only the *P. vivax* blood stage parasites, resulting in a short-term clinical cure, but these medicines do not eliminate the liver stage infection, leaving the patient at risk of later malaria illness relapses.
In a study conducted in Ethiopia in 2009, CQ and AL treatments both caused clinical cures of *P. vivax* malaria lasting up to three weeks in all patients; however, unacceptably high rates of recurrent *P. vivax* parasitemia were noted at 42 days of follow-up, with 41.6% (47/113) in the AL arm and 34% (34/107) in the CQ arm (unpublished data). Many of these recurrent malaria parasitemias were likely caused by relapses of dormant infections, although some probably were from new *P. vivax* re-infections. *P. vivax* infection is ideally treated with either CQ or AL resulting in a clinical cure, then with primaquine (PQ) resulting in a radical cure by eliminating the dormant liver hypnozoite forms. Primaquine radical cure is the standard of care in malaria-free areas such as the United States to prevent relapses and primaquine is the recommended WHO policy for adequate Pv treatment [2].

One study assessing the efficacy of CQ vs. AL both given with primaquine noted cure rates of 97.4% for AL vs. 100% for CQ at day 28 in Thailand [31]. In Ethiopia, the addition of primaquine to CQ decreased the cumulative incidence of therapeutic failure at day 28 by a life-table analysis method from 5.76% to 0.75% and the cumulative risk of relapse at day 157 by a life-table method from 61.8% to 26.3% [32].

Primaquine was previously widely used in Ethiopia according to national malaria treatment guidelines until the late 1990's. However, due to G6PD-related safety concerns that were mostly reported in other countries, primaquine use is now limited to Ethiopian health facilities with good laboratory capacity in non-malarious areas typically located above 2000m altitude. There is currently limited G6PD testing capacity in Ethiopia. The Ethiopian national malaria program is very interested in gathering credible evidence to support the scale up and safe deployment of primaquine for *P. vivax* treatment as the country moves towards malaria elimination.

In addition to assessing the efficacy of AL or CQ alone for *P. vivax* treatment, we propose to improve our understanding of the safety and the efficacy of primaquine use in G6PD non deficient patients in combination with these medications through a randomised, trial comparing the current practice of not using primaquine for most *P. vivax* cases with the WHO recommended standard therapy for *P. vivax* malaria (PQ 0.25mg/kg daily for 14 days).

OBJECTIVES

To assess the therapeutic efficacy of AL compared to AL+PQ and CQ compared to CQ+PQ for *P. vivax* infection and to determine the number of recurrent vivax episodes in patients receiving PQ compared to those who don't receive PQ.

Specific Objectives

Phase 1:

1) **Efficacy:** To measure the clinical and parasitological efficacy of AL, CQ, AL+PQ, or CQ+PQ for *P. vivax* infection during a 42-day follow-up period. (See Annex I for WHO definitions of *P. vivax* treatment failures)
2) **Safety:** To compare changes in hemoglobin concentration following vivax infection in patients receiving PQ versus patients who don't receive PQ.

Phase2:

1) **Efficacy:** To measure the clinical and parasitological efficacy of AL vs. AL+PQ, or CQ vs. CQ+PQ among patients $\geq$ 12 months of age suffering from uncomplicated vivax malaria by determining the number of recurrent vivax episodes (symptomatic as well asymptomatic) in patients receiving PQ versus patients who don't receive PQ during a 12 month follow up period.

STUDY DESIGN

This trial is a randomised, open label evaluation of clinical and parasitological responses to directly observed treatment for uncomplicated *P. vivax* malaria. Patients with uncomplicated *P. vivax* malaria infection who meet the study inclusion criteria will be enrolled, randomised, and treated on site with either AL or CQ with or without PQ, and monitored for 12 months. The drug administration will be directly observed. The follow-up will consist of a fixed schedule of check-up visits and corresponding clinical and laboratory examinations. On the basis of the results of these assessments, the number of adverse events and number of relapses will be compared between the study arms.

Study Sites

Evaluation sites in Oromia Regional State will be selected based on availability of adequate numbers of cases of symptomatic, uncomplicated *P. vivax* malaria infections, and willingness of the selected health care facility staff to participate in the study and to support the work with laboratory space. All sites will be public health centers operated by the Oromia Regional Health Bureau. We

have preliminary selected Bishoftu Malaria Control Center and Bulbula Health Center based on epidemiologic data, but we will continue to follow the malaria situation at these sites to determine the need for replacement sites from other districts and health facilities in Oromia Regional State in Ethiopia in order to obtain sufficient patient numbers for recruitment.

Drugs

All drugs will be GMP-certified and obtained from WHO.

Known efficacy and adverse effects of treatment drugs

- **Artemether - lumefantrine (Coartem or AL):** AL is currently the first line anti-malarial recommended by Ethiopian Ministry of Health for the treatment of uncomplicated falciparum infection, mixed Pf and *P. vivax* infections, and treatment of clinical malaria cases without laboratory confirmation which comprises of non-malaria cases, falciparum cases, and vivax cases. It is also an alternative medication for *P. vivax* infection when CQ is unavailable in Ethiopia. Adverse events are generally mild, most commonly gastrointestinal (GI) (vomiting and diarrhea) and hematologic (anemia and eosinophilia) [33]. Its safety was demonstrated in studies from Ethiopia and Uganda and in many other countries [34, 35].

- **Chloroquine (CQ):** Chloroquine, a 4-aminoquinoline, was recognized as an effective and safe antimalarial in 1946, and was the most widely used drug against malaria until significant resistance to chloroquine rendered it ineffective against *P. falciparum* by 1999. CQ is currently recommended by Ethiopian Ministry of Health for the treatment of *P. vivax* infection. Resistance to CQ has been described mostly in Southeast Asia in *P. vivax*, but available evidence shows relatively low levels of resistance in Ethiopia. Side effects include dizziness, skeletal muscle weakness, mild GI disturbances (nausea, vomiting, abdominal discomfort, and diarrhea), and pruritus [36].

- **Primaquine (PQ):** Primaquine is an 8-aminoquinoline and is effective against intrahepatic forms of all types of malaria parasite. It is used to provide radical cure of *P. vivax* and *P. ovale* malaria, in combination with a blood schizontocide such as CQ or AL for the erythrocytic parasites. The most important adverse effects are hemolytic anemia in patients with glucose-6-phosphate--dehydrogenase (G6PD) deficiency. In patients with the most common African variant of G6PD deficiency, the standard course of primaquine generally produces a benign self-limiting anemia. Therapeutic doses may also cause abdominal pain if administered on an empty stomach. Larger doses can cause nausea and vomiting. Methemoglobinaemia may occur. Other uncommon effects include mild anemia and leukocytosis. The severity of hemolytic anemia is related to primaquine dosing and the variant of the G6PD enzyme. Fortunately, primaquine is eliminated rapidly and so hemolysis is self-limiting provided no further drug is taken.

Drug Dosing and Regimens: All patients will be weighed to determine the accurate weight-based dose for both drugs.

- **Artemether-lumefantrine** (Coartem; Novartis) administered twice daily for three days as tablets containing 20 mg of artemether plus 120 mg of lumefantrine in a fixed dose combination at a dosage [37].

Weight (kg)	Age	Day 1		Day 2		Day 3	
		Morning	Evening	Morning	Evening	Morning	Evening
5–14	3mo–2yrs	1	1	1	1	1	1
15–24	3–7 yrs	2	2	2	2	2	2
25–34	8–10 yrs	3	3	3	3	3	3
> 35	10+ yrs	4	4	4	4	4	4

- **Chloroquine:** Total of 25mg base per kg over 3 days (10 mg base/kg on Days 1 and 2, and 5 mg base/kg on Day 3) [37]. 500 mg tablets and 10mg/ml syrup formulations.

Weight (kg)	Age	Day 1	Day 2	Day 3
5–6	<4 mo	¼ tablet (5 ml syrup)	¼ tablet (5 ml syrup)	⅛ tablet (2.5 ml syrup)
7–10	4–11 mo	¼ tablet (7.5 ml syrup)	¼ tablet (7.5 ml syrup)	¼tablet (5 ml syrup)
11–14	1–2 yrs	½ tablet (12.5 ml syrup)	½ tablet (12.5 ml syrup)	¼ tablet (7.5 ml syrup)
15–18	3–4 yrs	½ tablet (15 ml syrup)	½ tablet (15 ml syrup)	½ tablet (15 ml syrup)
19–24	5–7 yrs	¾ tablet (20 ml syrup)	¾ tablet (20 ml syrup)	½ tablet (15 ml syrup)
25–35	8–10 yrs	1¼ tablet	1¼ tablet	½ tablet
36–50	11–13 yrs	1½ tablet	1½ tablet	1 tablet
> 50	14+ yrs	2 tablet	2 tablet	1 tablet

- **Primaquine:** 0.25 mg/kg daily for 14 days will be administered on days 2-16 to those with a confirmed normal G6PD test by NADPH fluorescent spot test, which is the most commonly used test and is currently recommended by the International Committee for Standardization in Hematology.

Body weight (kg)	mg	Number of 15mg tablets
	based on 0.25mg/kg	PER DAY FOR 14 DAYS
5-14	1.25 to 3.5	¼
15-24	3.75 to 6	¼
25-34	6.25 to 8.5	½
35 -60 And more	8.75 to 15	1

Intervention or treatment

Antimalarial Therapy- All patients will be randomized to receive AL or CQ. Once G6PD results are available, those testing normal will be randomized to PQ or no PQ.. In Ethiopia G6PD deficiency is very rare (0.5-2.9%) [38]. All doses of CQ, PQ, and morning AL will be given under supervision by the staff. The evening dose of AL will be given to the patients to take at home. Children less than 5 years of age will be given an extra evening dose in case of vomiting. Patients will be observed for 60 minutes after treatment for adverse reactions or vomiting. Those patients vomiting their medication within the first 30 minutes will receive a repeat full dose, those vomiting from 30-60 minutes will receive half dose. This is standard practice for anti-malarial treatment.

Rescue Treatment

Patients failing in any arm within 14 days of treatment will be treated with quinine 10mg/kg/dose every 8 hours for 7 days as per National Guidelines [37]. This treatment will be administered orally unless the patient has persistent vomiting, in which case he/she will be referred to the nearest hospital. Patients presenting after 14 days of treatment will receive the same therapy.

Concomitant Treatment Using standard clinic procedures, clinic personnel will administer supportive treatment to patients as necessary:

- **Antipyretics** will be given for temperatures > 38°C as per national policy. All patients enrolled in the study will be given two additional doses of paracetamol for use at home. Patients/parents/guardians should be instructed in the use and application of tepid sponging and fanning as per national policy.
- **Ferrous Sulfate/ Folate** will be given to all children with hemoglobin <11 mg/dl as per Integrated Management of Childhood Illness (IMCI) Guidelines
- **Anti-helminthics** will be given to children with hemoglobin <11 mg/dl over one year of age as per IMCI Guidelines

<u>**Population**</u>

Description and source of population and catchment area

The population of interest consists of patients aged above 1 year living in malarious areas diagnosed with uncomplicated *P. vivax* malaria consulting at the health facility, who or whose parents or guardians give permission for study inclusion. Children cannot be excluded as they represent a high-risk population for malaria and it is imperative that the first-line antimalarials are effective in this group. For logistical reasons, only patients living near the enrolling health care facilities will be included (within 20 km), as we plan to visit the houses of those patients who do not return for scheduled follow-ups.

Initial screening *inclusion* criteria:

- Slide-confirmed infection with *P. vivax*
- Age > 1 year
- Lives within 20 km of the enrolling health facility

Inclusion criteria for enrollment:

1. Meet screening inclusion criteria
2. Weight ≥ 5.0 kg
3. Axillary temperature ≥ 37.5º C or history of fever during the previous 48 hours
4. Patient or caregiver agrees to all finger pricks and return visits.

Exclusion criteria for enrollment:

1. General danger signs or symptoms of severe malaria (see Annex II)
2. Signs or symptoms of severe malnutrition, defined as weight-for-age ≤ 3 standard deviations below the mean (NCHS/WHO normalized reference values; see Annex III)
3. Slide confirmed infection with any other *Plasmodium species.* besides *P. vivax* mono-infection
4. Acute anemia, defined as Hg < 8 g/dl
5. Known hypersensitivity to any of the drugs being evaluated
6. Presence of febrile conditions caused by diseases other than malaria
7. Serious or chronic medical condition by history (cardiac, renal, hepatic diseases, sickle cell disease, HIV/AIDS)
8. Pregnant or breastfeeding women.
9. History or hemolysis or severe anemia
10. Regular medication, which may interfere with antimalarial pharmacokinetics (Annex IV)

Sampling, sample size and statistical power

Sample size determination:

The design of the study is to compare the addition of PQ to AL or CQ alone. From our previous efficacy study, CQ alone showed a success rate of 68% at day 42 (null hypothesis). It will be assumed under the alternative hypothesis that the addition of PQ will decrease the chance of relapse by at least 60% resulting in a success rate of 87%. Group sample sizes of 97 in each group will achieve 90% power to detect a difference between group proportions of 19% using the two-sided Z-test with pooled variance test statistic. The significance level of the test was targeted at 0.05. Adjusting the alpha level to 0.025 for multiple comparisons will increase the sample size to 118 in each group. The AL arm showed even a lower efficacy at day 42 in our previous study of 58%. Using this lower success rate of 58% seen with AL at day 42, resulted in a smaller required sample size of 68. Taking into account 10-20% loss to follow-up at 42 days, a sample size of 120 subjects for each treatment was determined to be optimal.

SCREENING AND ENROLLMENT PROCEDURES

Screening Procedures for Enrollment

Screening for eligible patients at the two study sites will be done in the following fashion:

1. All patients presenting to the evaluation sites will be registered. At malaria treatment centers all patients will get a blood smear, whereas at health centers all patients presenting with fever will then get a blood smear. Patients with microscopy confirmed diagnosis of Pv malaria illness, age greater than 1 year, and living within 20 km of the health center will be screened for eligibility for enrollment. Those patients who for any reason are not enrolled into this study according to the screening and enrollment process will be treated promptly according to the current National Malaria Diagnosis and Treatment Guidelines [1].
2. If the patient meets the screening criteria and the patient or the parent or guardian asked for permission consents to enrollment, they will be assigned a consecutive, unique number that will become the patient's study number and will be used to identify all forms and blood samples from that patient. Enrollment will be conducted by a staff member not involved in the clinical assessment of the participant to ensure that consent is freely given.

3. A screening form containing demographic, clinical, and laboratory information will be started on each smear positive patient, thus allowing for appropriate tracking of each patient through the screening procedure.
4. The patient's body weight will be measured, using a hanging scale for weighing young children, or a floor scale for large children/ adults and recorded on the demographic form.

Enrollment Procedures

If a patient meets the initial screening criteria and permission to participate in the study is obtained, the patient will go through the enrolling process.

Clinical Evaluation:

1. Patient will be evaluated by clinical staff for signs of febrile illness other than malaria, (including but not limited to pneumonia, otitis media, tonsillitis, measles, chicken pox, abscesses) and signs of severe disease/ danger signs, as presence of any of these will exclude the patient from participating in the study. Patients found to have any of these conditions will be treated appropriately per National Guidelines [1], or referred to the nearest health center if seen at a malaria treatment center. A standardized form listing demographic and clinical information will be completed on each patient.

Laboratory Evaluations:

2. Urine pregnancy test will be conducted on all women aged 13-49 and anyone testing positive will be excluded.
3. Those enrolled will have a second finger prick performed to assess for the following:
    - Thin smear will be used to verify parasite species and thick smear to conduct a formal parasite count. Two qualified microscopists will read all the slides independently. Blood smears with discordant results (differences between the two microscopists in species diagnosis, in parasite density of > 50% or in the presence of parasites) will be re-examined by a third, independent microscopist, and parasite density will be calculated by averaging the two closest counts.
    - Hemoglobin measurement (HemoCue™ Hb 301HemoCue, Angelholm, Sweden) will be carried out and those with hemoglobin <8g/dL will be excluded.
    - Filter paper will be obtained for the purpose of genotyping to differentiate identical vs. different genotypes, potential molecular testing for resistance markers, and gametocyte testing. G6PD testing using the NADPH fluorescent spot test will be conducted at the end of each day. The NADPH fluorescent spot test is a qualitative test and those determined to be G6PD deficient will not be given primaquine.
4. Patients testing smear positive for Pv will receive AL, at one site and CQ at the other site. After G6PD testing results are available, the patients not found to be G6PD deficient will be randomized to either AL alone or AL+PQ at one study site and CQ alone or CQ+PQ at the other site. Those found to be G6PD deficient will remain in either the AL or CQ alone arms. The study will not be blinded, although microscopists reading blood films will not be aware of which arm a participant is from.
5. On day 0, each patient will receive the morning dose of treatment (AL or CQ) medication under direct observation and will be monitored for vomiting for 60 minutes. The patient or parent will be given the evening dose (for AL only) to administer at home. Each patient will return on days 1 and 2 (and up to 14 days for those in the PQ arms) for subsequent doses. Those in the primaquine arms will return on every second day on days 2-16 for directly observed therapy. Clinical reassessments will be made on days 1- 3, 7, 14, 21, 28, 35, 42, and then monthly (see Annex V and VI for a table of scheduled procedures).

    Participants will be given 35 Ethiopian Birr for each visit to cover their travel expenses (this is

enough to cover round-trip transportation by bus to the clinic for one adult; parent traveling with the enrolled child requiring their own seat (age >6 years) will receive 50 Birr). Patients will be advised to return on *any* day during the follow-up period if symptoms return and not to wait for scheduled visit days (although they will not be reimbursed for extra visits unless found to be legitimate visits). Patients who do not present to the facility for follow-up visits will be followed at home. This is standard for in vivo studies based on the WHO protocol, but is not the standard for routine care. Typically, for patients not enrolled in a study, the patients would be given all the doses of the medicine to administer at home, and no follow-up would be scheduled. The patient would be told to return if the child did not improve.

6. Blood films (thick and thin) for parasite count will be obtained and examined at each visit and on any other day if the patient spontaneously returns with fever or worsening symptoms.
7. Hemoglobin status will be measured on days 0, 3, 7, 14, 28, 42 and any other unscheduled visits.
8. **Drug level testing.** DBS will be collected on day 7 for lumefantrine plasma level [39], day of recurrent parasitemia for chloroquine levels [40], and day 7 and 14 for primaquine levels. Drug level testing involves liquid chromatography and these tests will be conducted at CDC Atlanta.
9. **Molecular testing.** A filter paper blood spot will be collected on days 0 and after day 3 in case of recurrent parasitemia for an array of parasite molecular testing including genotyping, resistance drug markers, parasite genome sequencing, and for gametocytes . In *P. falciparum* malaria primaquine accelerates clearance of gametocytes and artemisinin derivatives prevent gametocyte development. However, in *P. vivax* malaria effect of these drugs on gametocyte carriages remains unclear due to limited studies. Therefore, we will examine gametocytemia by blood smear and measuring Pvs25 using reverse –transcript quantitative PCR tool in the smear positive DBS from day 0 and follow up visits. Lastly, for any patients noted to have G6PD deficiency or a greater than 2.4g/dL drop in hemoglobin, their DBS sample will be tested for genetic variants of G6PD deficiency. The molecular testing will be conducted in Addis Ababa, Ethiopia, by Ethiopia Health and Nutrition Research Institute (EHNRI). If EHNRI cannot accomplish this in a timely manner, CDC Atlanta, will serve as a back-stop institution. All samples will have the study ID number and date of collection, but no other personal identifiers. The logbook that links the study ID number to the patient will be kept at the study site locked in a cabinet when not in use during the study enrollment period. After the completion of the study, this logbook will be stored in a locked cabinet in Addis Ababa at ICAP's office. DBSs will not be used for non-malaria-related tests including HIV.

FOLLOW-UP PROCEDURES

Study Instruments

Standard operating procedures and structured forms for clinical history, physical assessment, and data collection in English will be used by each study site. All study staff are familiar with English and health systems data collection in the country are routinely done in English.

Active Surveillance

Study participants will be seen by a health care worker/investigator on days 0, 1, 2, 3, 7, 14, 21, 28, 35, 42, and thereafter 1 time per month for 12 months. A blood slide will be collected at every visit to check for parasitaemia, whether symptomatic or asymptomatic. In addition, patients will be contacted by phone weekly for 12 months to detect fever episodes and to stay informed about migration.

Passive Surveillance
Patients will be advised to return on any day during the 12 months follow-up period if symptoms return and not to wait for the next scheduled visit day. In particular, parents or guardians should be instructed to bring children to the center at any time if they show any sign of danger (unable to drink or breastfeed, vomiting everything, presenting with convulsions, lethargic or unconscious, unable to sit or stand, presenting with difficult breathing), if they are still sick or if there is any cause for worry. The participants will receive a study card to alert the health care provider to the participation status. Participants should continue to receive the allocated treatment for the duration of the follow-up period.

The basic follow-up schedule is summarized in Annex V and VI. A case report form and an adverse event report form will be used to record the general information and clinical observations on each patient enrolled into the study. Recurrent malaria episodes will be entered in the standard reporting form. The appointment schedule will be clearly explained, and a follow-up card with a personal identification number will be provided in addition to the patient ID card.

All reasonable efforts will be made to find defaulters to ensure complete treatment and follow-up. While patients are encouraged to return on their own for scheduled follow-up visits, it is essential that provisions be made ahead of time for locating patients at home if they do not attend as requested. This requires obtaining detailed directions to the home during enrolment, and study team members familiar with the community will be responsible for home visits and means of transport for the patients. The schedule of treatment and follow-up examinations given in this protocol must be followed to ensure data integrity.

END-POINTS

Outcomes and minimal differences

Efficacy end points
The primary outcome of this study will be to estimate the efficacy of AL compared to AL+PQ, and CQ compared to CQ+PQ against vivax malaria at Day 28/42.

In addition, any episode of vivax parasitemia during the one year follow-up following parasite clearance will be considered a recurrence. Each recurrence will be treated in the same fashion as the initial episode, the patient remains in the treatment arm he or she has been randomised to for the duration of the follow-up period.

Safety end points
Change in hemoglobin concentration within 7 days of start of treatment in PQ compared to the no PQ arm. Hemoglobin concentration will be assessed on day 0, 3, 7, 14, 28, 42. All adverse events will be documented. The proportion of participants with solicited and unsolicited adverse events, overall and specific, will be compared between treatment arms. Events will be analysed in relation to time of the drug administration.

The classification of treatment outcomes will be based on an assessment of the parasitological and clinical outcome of antimalarial treatment according to the latest guidelines from WHO [41].

Definition of Treatment Outcomes for *P. vivax* infections, WHO 2002 [42]

Outcomes defined as:

For Primaquine:

- Number of vivax episodes following initial parasite clearance during the follow-up period.

For artemether-lumefantrine or chloroquine:

Early treatment failure

- danger signs or severe malaria on day 1, 2 or 3 in the presence of parasitaemia;
- parasitaemia on day 2 higher than on day 0, irrespective of axillary temperature;
- parasitaemia on day 3 with axillary temperature ≥ 37.5 ºC;
- parasitaemia on day 3 ≥ 25% of count on day 0.

Late treatment failure

Late clinical failure

- danger signs or severe malaria in the presence of parasitaemia on any day between day 4 and day 28 (day 42) in patients who did not previously meet any of the criteria of early treatment failure;
- presence of parasitaemia on any day between day 4 and day 28(day 42) with axillary temperature ≥ 37.5 ºC (or history of fever) in patients who did not previously meet any of the criteria of early treatment failure

Late parasitological failure

- presence of parasitaemia on any day between day 7 and day 28 (day 42) with axillary temperature < 37.5 ºC in patients who did not previously meet any of the criteria of early treatment failure or late clinical failure

Adequate clinical and parasitological response

- absence of parasitaemia on day 28 (day 42), irrespective of axillary temperature, in patients who did not previously meet any of the criteria of early treatment failure, late clinical failure or late parasitological failure

P. vivax infections that recur after drug treatment can have 1 of 3 etiologies: 1) recrudescence, 2) reinfection, or 3) relapse arising from the dormant liver stage (hypnozoites). In order to differentiate the recurrent parasitemias as identical vs. different from the original infection, a genotypic analysis will be performed by PCR including characterizing microsatellite markers to assess recrudescence from reinfection and for drug resistance markers. Molecular testing for gametocytes will also be conducted on any smear positive days. Those who fail in the chloroquine arm will have CQ levels tested from the already collected filter paper. Lumefantrine level on day 7 will be measured for those receiving AL treatment from collected filter papers. Patients noted to have G6PD deficiency by the fluorescent spot test and those with hemoglobin drop of >2.4g/dL will undergo additional G6PD genotyping.

Valid end-points include treatment failure, completion of the follow-up period without treatment failure, loss to follow-up, withdrawal from study (voluntary or involuntary, and protocol violation). Loss to follow-up occurs when, despite all reasonable effort, an enrolled patient who does not attend the scheduled visits, cannot be found. Patients who withdraw from the study during the treatment period (days 0-2) will be treated with CQ according to the national policy. They will be referred back to the clinic to continue with care.

Criteria for withdrawal/protocol violation

Patients meeting any of the following criteria will be withdrawn:

- Withdrawal of consent
- Failure to complete the treatment
- Vomiting both initial and replacement doses at any single time in the treatment (i.e. If the patient vomits both attempts to administer the morning dose that would require withdrawal; however, vomiting the initial morning dose but not the morning replacement dose would not require withdrawal), persistent vomiting
- Severe side-effects necessitating hospitalization
- Progression to severe malaria
- Occurrence during the follow-up of concomitant disease that would interfere with a clear classification of the treatment outcome
- Antimalarial (or antibiotics with antimalarial activity) treatment administered by a third party or self-medication with antimalarial (or antibiotics with antimalarial activity- Annex IV) assessed by asking participants or their caretaker at follow-up visits
- Erroneous inclusion of a patient outside of the inclusion/exclusion criteria
- Misclassification of a patient due to a laboratory error (parasitemia) leading to the administration of the rescue treatment

Patients who are withdrawn will be followed up if possible and will be censored or excluded from the analysis. The reasons for discontinuation or protocol violation will be recorded on the case report form.

Training for personnel: Clinicians and laboratory personnel are trained and licensed in Ethiopia. In addition, they will receive approximately 1 week additional training to review the protocol of this study. In addition to technical aspects of specific laboratory techniques and clinical assessments, training will stress the importance of confidentiality, and appropriate techniques for obtaining informed consent. Continued appropriate processes with regard to informed consent will be reinforced by intermittent observation by the supervisor. The staff will include 1-2 laboratory technicians for microscopy, and 1-2 staff persons for enrollment and clinical assessments.

DATA HANDLING AND ANALYSIS

Data analysis plan, including statistical methodology and planned tables and figures
SAS 9.3 (Cary, NC) as well as the WHO drug efficacy tools provided at http://www.who.int/malaria/toolsformonitoring.html will be used for data management and analysis. Data will be analyzed using two methods: **per protocol analysis**, where patients who were withdrawn from the study or who were lost to follow-up are excluded, and **survival analysis**, where all enrolled patients are included in the analysis until the last day before drop-out. In addition to voluntary withdrawals or treatment non-compliance, patients will be considered for the analysis as withdrawn if the PCR results are unclassifiable.

The final analysis will include:

- A description of the patients screened, and the distribution of the reasons for exclusion.
- A description of the patients included in the study.
- The proportion of patients lost to follow-up or withdrawn and the distribution of reasons for withdrawal.
- The proportion of treatment failures in each Pv arm at day 28 and day 42 with 95% confidence intervals using both methods of analysis.
- Frequency of side effects, severe malaria, anemia, and hospitalizations by treatment arm.
- Comparison in absolute means and relative changes in hemoglobin concentration during follow-up after treatment
- Comparison of number of recurrent parasitological vivax episodes after initial parasite episode.

Data collection: Data will be collected and recorded at the health facility by one of the trained staff members. All data entry forms will be checked for completeness. Laboratorians may complete brief reports on laboratory findings to share with clinicians who will transfer these results to the case report form of the appropriate patient, the laboratory results will be entered into an Excel database.

Information management and analysis software: Microsoft Excel (Redmond, Washington) will be used for double entry of laboratory data and clinical data. SAS 9.3 (Cary, NC) will be used for data analysis.

Data entry, editing, handling, storage and disposition: Some open-ended data including the names of specific drugs or diagnoses will be coded prior to data entry. A list of standard codes for these fields will be developed by hand once collection of data is complete. All forms will then be coded and checked by 2 different team members.

Data will be double entered using Microsoft Excel (Redmond, Washington). Systematic routines will be developed to check for data entry discrepancies and range and consistency. All discrepancies will be resolved by reference to the original checked data collection forms. The final data set will be transformed to SAS for analysis and will no longer contain any personal identifying information.

Original data collection forms will be handled only by staff members and kept under locked storage until completely coded, checked and transported for data entry. Once data entry and cleaning are

complete the original forms will be stored under lock and key at ICAP until final analyses and reports have been prepared. They will then be destroyed.

Storage of specimens: Specimens obtained in this study will be saved only for the duration of molecular testing and drug testing of treatment failures and then destroyed. No samples will be stored. Specimens will not be tested for HIV.

Quality control/ assurance: Site supervisors will check data collection forms at the end of follow-up for completeness and accuracy of recording.

Bias in data collection, measurement and analysis: Some bias may be introduced through the fact that both treating clinicians and parents will be aware of which treatment a subject received; as much as possible we will rely on objective measures of improvement (fever resolution, malaria parasitemia) which will be unaffected by knowing the treatment allocation. In addition, the microscopists reading the slides will be blinded to the treatment allocation. Other biases in data collection will be minimized by use of standardized data collection instruments.

Limitations: Results from this study may not be applicable to other ACTs or other Pv-endemic areas with different resistance patterns. The health facilities will not be chosen randomly and thus may not be representative of our target population.

HANDLING OF UNEXPECTED OR ADVERSE EVENTS

Response to new or unexpected findings

All treatment failures will be treated with quinine as per the National Guidelines. If sufficient number of malaria cases are presenting to the clinic but enrolment is insufficient, the supervisor will need to determine at what point the problem with enrollment is occurring, and take steps needed to rectify the problem. If the problem is not remedied, the supervisor will report the poor enrollment to the investigators at CDC, Atlanta, and steps will be taken to either rectify the problem or to find a new site.

Identifying, managing and reporting adverse events

We will be observing all the children for an hour following the ingestion of every dose of antimalarial. At every visit, patients will complete an adverse events history.

In the event of mild adverse reactions, it will be noted in the subjects case report form that these occurred; no further action will be taken by staff members, except in the case of vomiting, in which case the treatment medication may need to be re-administered. In the event of significant vomiting, the patient will be referred to the district hospital for further management. In the case of any severe adverse reaction (difficulty breathing, convulsions, and change in mental status) subjects will be referred to the district hospital for management. Transportation to the hospital will be provided.

Any adverse event which is severe, unexpected, and possibly related to the study, as well as any unanticipated problems involving risks to subjects or others and any occurrences of serious or continuing non-compliance will be reported to the CDC IRB according to CDC policy on incident reporting.

EMERGENCY CARE
Any patient with treatment failure or recrudescence will receive quinine 10mg/kg/dose every 8 hours for 7 days. This will be given orally unless the patient has persistent vomiting, in which case the patient will be referred to the nearest hospital to receive IM/IV therapy.

Patients with signs of severe malaria (altered mental status, coma, convulsions, hemoglobin <5g/dl, respiratory distress, circulatory collapse, or abnormal bleeding), persistent vomiting or severe side effects will be hospitalized and receive parenteral therapy with artesunate (or quinine if not available) and relevant supportive treatments, according to National Guidelines.

Any participant who develops urticaria or difficulty breathing following administration of the treatment drug will be treated with diphenhydramine or chlorpheniramine and will be withdrawn from the study. In addition, patient and or their parents, caretakers and family members will be counseled that the patient should not receive additional doses of the antimalarial they were given. These patients will be treated with oral quinine for seven days as per the rescue protocol.

Adult patients and parents with children in the study will be encouraged to return to the clinic for any fever or other signs of illness on any day during the follow-up period, allowing for early detection and treatment of illness before it becomes severe. Any hospitalization which occurs during the follow-up period will be paid for by the study.

ETHICAL ISSUES

Consent Process
Staff members will fully explain the assessments to the participants and or their caretakers and ask for their consent/assent to participate. The written consent documents in English are attached as Annex VII. These will be translated in Afaan Oromoo and Amharic and then back translated to verify accuracy. These will be read to participants and or their caretakers in their respective local language. If a participant or parent/ guardian are illiterate, the consent form will be read to them in their respective local language and a thumbprint will be accepted as a legally effective signature. Consenting participants and or their caretakers will be advised that they are free to decline any question or procedure and that they may terminate their participation at any time without loss of any benefits.

Purpose: The clinical efficacy of the first-line antimalarials CQ and AL will be assessed using a WHO standardized procedure. The addition of PQ to CQ or AL, the WHO recommended regimen, will also be assessed. These results will be used to assist Ethiopia in re-assessing their national malaria drug policies and investigating therapeutic options for the future.

Procedures: During the course of the study, patients will receive an antimalarial and will undergo ~20 finger pricks to allow monitoring of parasitemia and anemia. Clinical assessments will be made on days 0, 1- 3, 7, 14, 21, 28, 35, 42, and then monthly for 1 year.

Duration of participant involvement: Patients will be involved in the study from day of enrollment (Day 0) until their 1 year follow-up.

Experimental products or procedures: No experimental therapies, products, or procedures will be used in this study. The use of AL for the treatment for Pv is common practice and WHO recommended practice in countries that have adopted AL for first-line treatment of Pf. Primaquine

is a standard component of therapy for Pv malaria illness to prevent relapses. The addition of PQ to a blood schizonticide (CQ or AL) is the WHO recommended regimen for Pv.

RISKS

There is minimal physical and psychological risk associated with this study. The patient may experience a brief moment of physical discomfort and/or fear during the finger prick.

There is minimal risk associated with taking AL, CQ, or PQ. All of the drugs are recommended therapies in Ethiopia, have been widely used, and have been found to be safe (excluding those with G6PD deficiency for PQ). Studies of all of these combinations have reported only mild or moderate adverse events, including dizziness, headache, anorexia, nausea and abdominal pain, noting that these events may also be attributable to malaria. The use of PQ in those without G6PD deficiency is well-tolerated with mild GI discomfort when taken on an empty stomach. The protocol screens for G6PD deficiency, identifying those at hemolytic risk from primaquine therapy, and ensures those that are deficient are not given primaquine.

There are no social risks associated with this protocol. Malaria is a widely recognized disease in Ethiopia, and seeking treatment is socially acceptable. There are no economic risks associated with this study. Patients will be assessed and treated free of charge, and families will be given a stipend of 35 Ethiopian Birr (50Birr for those accompanied by a parent) per visit to cover their travel expenses. Any hospitalization that results from this study or during the follow-up period will be paid for.

METHODS TO MINIMIZE RISKS

In order to minimize risks to patients, subjects will be followed closely for signs of recurrent parasitemia and hemolysis, with clinical and parasitological exams on days 1- 3, 7, 14, 21, 28, 35, 42, and monthly thereafter for one year. In addition, patients will be encouraged to return on any other day with signs of illness, fever, or dark urine. All patients will be observed closely for an hour following the first dose of medication. Any patient who develops urticaria or difficulty breathing following administration of the treatment drug will be treated with diphenhydramine or chlorpheniramine and will be withdrawn from the study. In addition, participants will be counseled that they should not take additional doses of other antimalarials (Annex IV).

ANTICIPATED BENEFITS

This study is of direct benefit to participants. All patients will be treated for their initial parasitemia and followed to confirm parasite clearance. Any patient failing treatment with the treatment drug regimen will be treated with quinine, another standard antimalarial medication. At each visit, the patient, parent or guardian will be informed as to the status of their or their child's health, and the procedures and/or treatments that will occur during that visit and have the right to refuse a procedure or treatment or request transfer to the regular clinic and withdrawal from the study. Patients with malaria at government clinics receive for free whatever treatment is available in stock at the clinic. Although AL and CQ is the national policy, it is not always available in government clinics. Those patients assigned to the PQ arm will be given anti-relapse therapy which is currently not commonly practiced in Ethiopia.

The findings from the study will assist decision making to improve prevention and treatment of malaria in Ethiopia. With universal access to AL in Ethiopia since 2004 and the long-term use of chloroquine for Pv, monitoring for resistance to both these first-line drugs will be paramount. As a country pursuing elimination, effective treatment of Pv with PQ to prevent frequent relapses will provide both hematologic benefit for patients and decreased transmission in the community.

RISK/BENEFIT RATIO
This study has minimal risks and significant benefit to the enrolled subjects.

VULNERABLE POPULATIONS
Children- This minimal risk study includes children. Because this group has minimal natural immunity, this is the group most at risk for severe malaria, so any treatment must be efficacious in this age group. Adequate provisions are made for soliciting the permission of a parent or guardian.

Non-English speaking participants- It is imperative to carry out this study in the areas in which malaria is endemic; therefore, it is unavoidable that most participants will be non-English speaking. All consent forms have been translated into Afaan Oromoo and Amharic and back translated into English for accuracy; all explanations of the study procedures and clinical findings will be carried out in the patient's local language by local staff member.

INFORMED CONSENT/ PARENTAL PERMISSION PROCESS/ DOCUMENTATION OF INFORMED CONSENT
Permission will be obtained from parent/ guardian bringing the child or the adult him/herself. Consent, and assent for children 7-17 years of age, will be appropriately documented prior to each patient's enrollment. As this study is minimal risk, permission from only one parent will be required. A staff member will read the form to the patient and or parent and give them a chance to ask questions. Patients and/or parents who are able to sign their name will be asked to sign the consent form. For non-literate persons, a thumbprint will be obtained in lieu of a signature. Children age 7-17 years of age will have a simplified assent form read to them. Details about the study and its benefits and potential risk will be explained to the patients in the language in which they are most fluent. All information regarding the patients will remain confidential to the extent legally possible. Unique numerical identifiers will be used for data entry.

JUSTIFICATION FOR WAIVER/ ALTERATION OF INFORMED CONSENT
No justification for waiver or alteration of informed consent is being sought.

There is a large illiterate population in Ethiopia; thus there is a high likelihood that parents may be unable to sign their names. In this case, permission will be documented with a thumbprint as a legally effective signature in Ethiopia, following thorough explanation of the study and its benefits and potential risk to the patients and or parents/guardians in the language in which they are most fluent. A witness who is not involved with enrolling the patients and literate will witness and sign the informed consent.

ASSENT IMPLEMENTATION PROCESS/ DOCUMENTATION OF ASSENT
Assent for children age 7-17 years of age will be sought.

PROTECTION OF PRIVACY AND CONFIDENTIALITY
Privacy of individual: Examinations will take place in a private room. Individual data will be reported to the participants and to relevant staff members, and will be recorded in case reporting forms.

Confidentiality of data: All screening forms and case record forms will be kept in a secured location with access limited to authorized staff members. Unique numerical identifiers will be used for the computer-based data entry and blood samples. Publications will contain only aggregate data. No identifying information will be included.

EXTRA COSTS TO PARTICIPANTS

Participants will not incur extra costs through their participation in this study. All examinations, laboratory testing, and medications will be provided free. Participants will be reimbursed for their travel costs. Their participation in the study will require additional visits, so there will be an extra time cost associated with participation.

REIMBURSEMENTS OR INCENTIVES

Patients will be reimbursed 35 Ethiopian Birr (50 Birr those children age>6 years accompanied by a parent) per visit for their travel expenses only to minimize loss to follow-up and to prevent patients from incurring extra expenses in travel to and from the clinic for the purpose of participating in the study. The travel reimbursement will be given to each participant even if they are in the same family.

DISSEMINATION, NOTIFICATION AND REPORTING OF RESULTS

Clinical trial registration

The clinical trial registration will be done by the investigators through the website www.clinicaltrials.gov.

Notifying participants of their individual results

Participants and their parents will be informed of the results of the malaria smear, hemoglobin level, G6PD status, and clinical assessments at the time of enrollment and at each follow-up visit. Filter paper analysis will be completed only after the study is completed at a reference laboratory. Participants will not be informed of their individual results from these tests.

Notifying participants of findings: Participants will not be directly notified of the study findings.

Anticipated products or inventions resulting from the study and their use

All results will be disseminated to the national malaria control programs and other appropriate bodies within the Ethiopian Government including the health centers where the studies will be conducted. We anticipate that the results of this study will be used to inform future changes to the National Malaria Diagnosis and Treatment Guidelines of Ethiopia. In addition, it is anticipated that the evaluators will develop abstracts for presentation at scientific meetings. Finally, one or more detailed manuscripts will be prepared for publication in a peer-reviewed scientific journal.

Disseminating results to public

The Ethiopian FMOH regularly disseminates key findings to the general public. This occurs through press releases and by including media reporters in data dissemination workshops with local leaders at the conclusion of each major activity, or sooner as important findings become available. Findings from this proposed study that may be of value to the general public can be disseminated through these existing mechanisms.

Scientific Peer Review

The results from this study will be published in at least one summary paper which will be submitted to a peer-review journal. In addition, additional abstracts may be developed from the results of this study.

Conflicts of Interest

We have no conflicts of interest.

REFERENCES

1. Ethiopia Federal Ministry of Health, *National Malaria Guidelines Third Edition*, M.o. Health, Editor 2012: Addis Ababa, Ethiopia. Available at link: http://www.moh.gov.et/English/Resources/Documents/National%20malaria%20guidelines_2012.pdf
2. WHO, *Guidelines for the treatment of malaria Second edition*, 2010, World Health Organization: Geneva.
3. Ethiopia Federal Ministry of Health, *Health and Health Related Indicators report EC 2002*, 2010, Ethiopia Federal Ministry of Health: Addis Ababa.
4. Nigatu, W., M. Abebe, and A. Dejene, *Plasmodium vivax and P. falciparum epidemiology in Gambella, south-west Ethiopia.* Trop Med Parasitol, 1992. **43**(3): p. 181-5.
5. Olana, D., et al., *Malaria, Oromia regional state, Ethiopia, 2001-2006.* Emerg Infect Dis, 2011. **17**(7): p. 1336-7.
6. Ramos, J.M., F. Reyes, and A. Tesfamariam, *Change in Epidemiology of Malaria Infections in a Rural Area in Ethiopia.* J Travel Med, 2005. **12**: p. 155-156.
7. Fogh, S., S. Jepsen, and R. Mataya, *Chloroquine-resistant Plasmodium falciparum malaria from Kenya.* Trans R Soc Trop Med Hyg, 1979. **73**: p. 228-229.
8. Alene, G.D. and S. Bennett, *Chloroquine resistance of Plasmodium falciparum malaria in Ethiopia and Eritrea.* Trop Med Int Health, 1996. **1**(6): p. 810-5.
9. Jima, D., et al., *Efficacy of sulfadoxine-pyrimethamine for the treatment of uncomplicated falciparum malaria in Ethiopia.* East Afr Med J, 2005. **82**(8): p. 391-5.
10. Kassa, M., et al., *Development of resistance by Plasmodium falciparum to sulfadoxine/pyrimethamine in Amhara Region, Northwestern Ethiopia.* Ethiop Med J, 2005. **43**(3): p. 181-7.
11. Ethiopia Federal Ministry of Health., *Malaria: Diagnosis and Treatment Guidelines for Health Workers in Ethiopia 2nd Edition*, 2004, Federal Democratic Republic of Ethiopia, Ministry of Health: Addis Ababa.
12. Rieckmann, K.H., D.R. Davis, and D.C. Hutton, *Plasmodium vivax resistance to chloroquine?* Lancet, 1989. **2**(8673): p. 1183-4.
13. Baird, J.K., *Resistance to therapies for infection by Plasmodium vivax.* Clin Microbiol Rev, 2009. **22**(3): p. 508-34.
14. Baird, J.K., et al., *Treatment of chloroquine-resistant Plasmodium vivax with chloroquine and primaquine or halofantrine.* J Infect Dis, 1995. **171**(6): p. 1678-82.
15. Baird, J.K., et al., *Whole blood chloroquine concentrations with Plasmodium vivax infection in Irian Jaya, Indonesia.* Am J Trop Med Hyg, 1997. **56**(6): p. 618-20.
16. Ratcliff, A., et al., *Therapeutic response of multidrug-resistant Plasmodium falciparum and P. vivax to chloroquine and sulfadoxine-pyrimethamine in southern Papua, Indonesia.* Trans R Soc Trop Med Hyg, 2007. **101**(4): p. 351-9.
17. Sumawinata, I.W., et al., *Very high risk of therapeutic failure with chloroquine for uncomplicated Plasmodium falciparum and P. vivax malaria in Indonesian Papua.* Am J Trop Med Hyg, 2003. **68**(4): p. 416-20.
18. Genton, B., et al., *Parasitological and clinical efficacy of standard treatment regimens against Plasmodium falciparum, P. vivax and P. malariae in Papua New Guinea.* P N G Med J, 2005. **48**(3-4): p. 141-50.
19. Guthmann, J.P., et al., *Plasmodium vivax resistance to chloroquine in Dawei, southern Myanmar.* Trop Med Int Health, 2008. **13**(1): p. 91-8.
20. Phan, G.T., et al., *Artemisinin or chloroquine for blood stage Plasmodium vivax malaria in Vietnam.* Trop Med Int Health, 2002. **7**(10): p. 858-64.
21. Taylor, W.R., et al., *Assessing drug sensitivity of Plasmodium vivax to halofantrine or choroquine in southern, central Vietnam using an extended 28-day in vivo test and polymerase chain reaction genotyping.* Am J Trop Med Hyg, 2000. **62**(6): p. 693-7.
22. Kurcer, M.A., Z. Simsek, and Z. Kurcer, *The decreasing efficacy of chloroquine in the treatment of Plasmodium vivax malaria, in Sanliurfa, south-eastern Turkey.* Ann Trop Med Parasitol, 2006. **100**(2): p. 109-13.
23. Kurcer, M.A., et al., *Efficacy of chloroquine in the treatment of Plasmodium vivax malaria in Turkey.* Ann Trop Med Parasitol, 2004. **98**(5): p. 447-51.
24. Soto, J., et al., *Plasmodium vivax clinically resistant to chloroquine in Colombia.* Am J Trop Med Hyg, 2001. **65**(2): p. 90-3.
25. de Santana Filho, F.S., et al., *Chloroquine-resistant Plasmodium vivax, Brazilian Amazon.* Emerg Infect Dis, 2007. **13**(7): p. 1125-6.
26. Tulu, A.N., et al., *Failure of chloroquine treatment for malaria in the highlands of Ethiopia.* Trans R Soc Trop Med Hyg, 1996. **90**(5): p. 556-7.
27. Ketema, T., et al., *Chloroquine-resistant Plasmodium vivax malaria in Serbo town, Jimma zone, south-*

west Ethiopia. Malar J, 2009. **8**: p. 177.
28. Teka, H., et al., *Chloroquine-resistant Plasmodium vivax malaria in Debre Zeit, Ethiopia.* Malar J, 2008. **7**: p. 220.
29. Yohannes, A.M., et al., *Confirmed vivax resistance to chloroquine and effectiveness of artemether-lumefantrine for the treatment of vivax malaria in Ethiopia.* Am J Trop Med Hyg, 2011. **84**(1): p. 137-40.
30. Ketema, T., K. Getahun, and K. Bacha, *Therapeutic efficacy of chloroquine for treatment of Plasmodium vivax malaria cases in Halaba district, South Ethiopia.* Parasit Vectors, 2011. **4**: p. 46.
31. Krudsood, S., et al., *Clinical efficacy of chloroquine versus artemether-lumefantrine for Plasmodium vivax treatment in Thailand.* Korean J Parasitol, 2007. **45**(2): p. 111-4.
32. Yeshiwondim, A.K., et al., *Therapeutic efficacy of chloroquine and chloroquine plus primaquine for the treatment of Plasmodium vivax in Ethiopia.* Acta Trop, 2010. **113**(2): p. 105-13.
33. Falade, C., et al., *Efficacy and safety of artemether-lumefantrine (Coartem) tablets (six-dose regimen) in African infants and children with acute, uncomplicated falciparum malaria.* Trans R Soc Trop Med Hyg, 2005. **99**(6): p. 459-67.
34. Jima, D., et al., *Safety and efficacy of artemether-lumefantrine in the treatment of uncomplicated falciparum malaria in Ethiopia.* East Afr Med J, 2005. **82**(8): p. 387-90.
35. Maiteki-Sebuguzi, C., et al., *Safety and tolerability of combination antimalarial therapies for uncomplicated falciparum malaria in Ugandan children.* Malar J, 2008. **7**: p. 106.
36. Baird, J.K., *Effectiveness of antimalarial drugs.* N Engl J Med, 2005. **352**(15): p. 1565-77.
37. Ethiopia Federal Ministry of Health, *Malaria: Diagnosis and Treatment Guidelines for Health Workers in Ethiopia*, 2004.
38. Group, W.W., *Glucose-6-phosphate dehydrongenase deficiency.* Bull World Health Organ, 1989. **67**(6): p. 601-611.
39. Blessborn, D., et al., *Development and validation of an automated solid-phase extraction and liquid chromatographic method for determination of lumefantrine in capillary blood on sampling paper.* J Pharm Biomed Anal, 2007. **45**(2): p. 282-7.
40. Patchen, L.C., et al., *Analysis of filter-paper-absorbed, finger-stick blood samples for chloroquine and its major metabolite using high-performance liquid chromatography with fluorescence detection.* J Chromatogr, 1983. **278**(1): p. 81-9.
41. WHO, *Methods for Surveillance of Antimalarial Drug Efficacy*, 2009, World Health Organization: Geneva.
42. WHO, *Monitoring Antimalarial Drug Resistance: Report of a WHO consultation*, 2002, World Health Organization: Geneva.
43. *Severe falciparum malaria. World Health Organization, Communicable Diseases Cluster.* Trans R Soc Trop Med Hyg, 2000. **94 Suppl 1**: p. S1-90.

ANNEX

ANNEX I *P. vivax* treatment outcomes

For Primaquine:

- Number of vivax episodes following initial parasite clearance during the follow-up period.

For artemether-lumefantrine or chloroquine:

Early treatment failure

- danger signs or severe malaria on day 1, 2 or 3 in the presence of parasitaemia;
- parasitaemia on day 2 higher than on day 0, irrespective of axillary temperature;
- parasitaemia on day 3 with axillary temperature ≥ 37.5 ºC;
- parasitaemia on day 3 ≥ 25% of count on day 0.

Late treatment failure

Late clinical failure

- danger signs or severe malaria in the presence of parasitaemia on any day between day 4 and day 28 (day 42) in patients who did not previously meet any of the criteria of early treatment failure;
- presence of parasitaemia on any day between day 4 and day 28(day 42) with axillary temperature ≥ 37.5 ºC (or history of fever) in patients who did not previously meet any of the criteria of early treatment failure

Late parasitological failure

- presence of parasitaemia on any day between day 7 and day 28 (day 42) with axillary temperature < 37.5 ºC in patients who did not previously meet any of the criteria of early treatment failure or late clinical failure

Adequate clinical and parasitological response

- absence of parasitaemia on day 28 (day 42), irrespective of axillary temperature, in patients who did not previously meet any of the criteria of early treatment failure, late clinical failure or late parasitological failure

ANNEX II Severe malaria definitions

Definition of severe malaria [43]

Severe manifestations of malaria in adults and children

**Prognostic value**[a]			**Frequency**[a]	
Children	Adults		Children	Adults
Clinical Manifestations				
+	(?)[b]	Prostration	+++	+++
+++	+	Impaired Consciousness	+++	++
+++	+++	Respiratory Distress (acidotic breathing)	+++	+
+	++	Multiple convulsions	+++	+
+++	+++	Circulatory collapse	+	+
+++	+++	Pulmonary edema	+/–	+
+++	++	Abnormal bleeding	+/–	+
++	+	Jaundice	+	+++
+	+	Hemoglobinuria	+/–	+
Laboratory findings				
+	+	Severe anemia	+++	+
+++	+++	Hypoglycemia	+++	++
+++	+++	Acidosis	+++	++
+++	+++	Hyperlactatemia	+++	++
+/–	++	Hyperparasitemia	++	+
++	++	Renal Impairment	+	+++

[a]On a scale from + to +++; +/– indicates infrequent occurrence
[b]Data not available

ANNEX III Weight for Height Chart

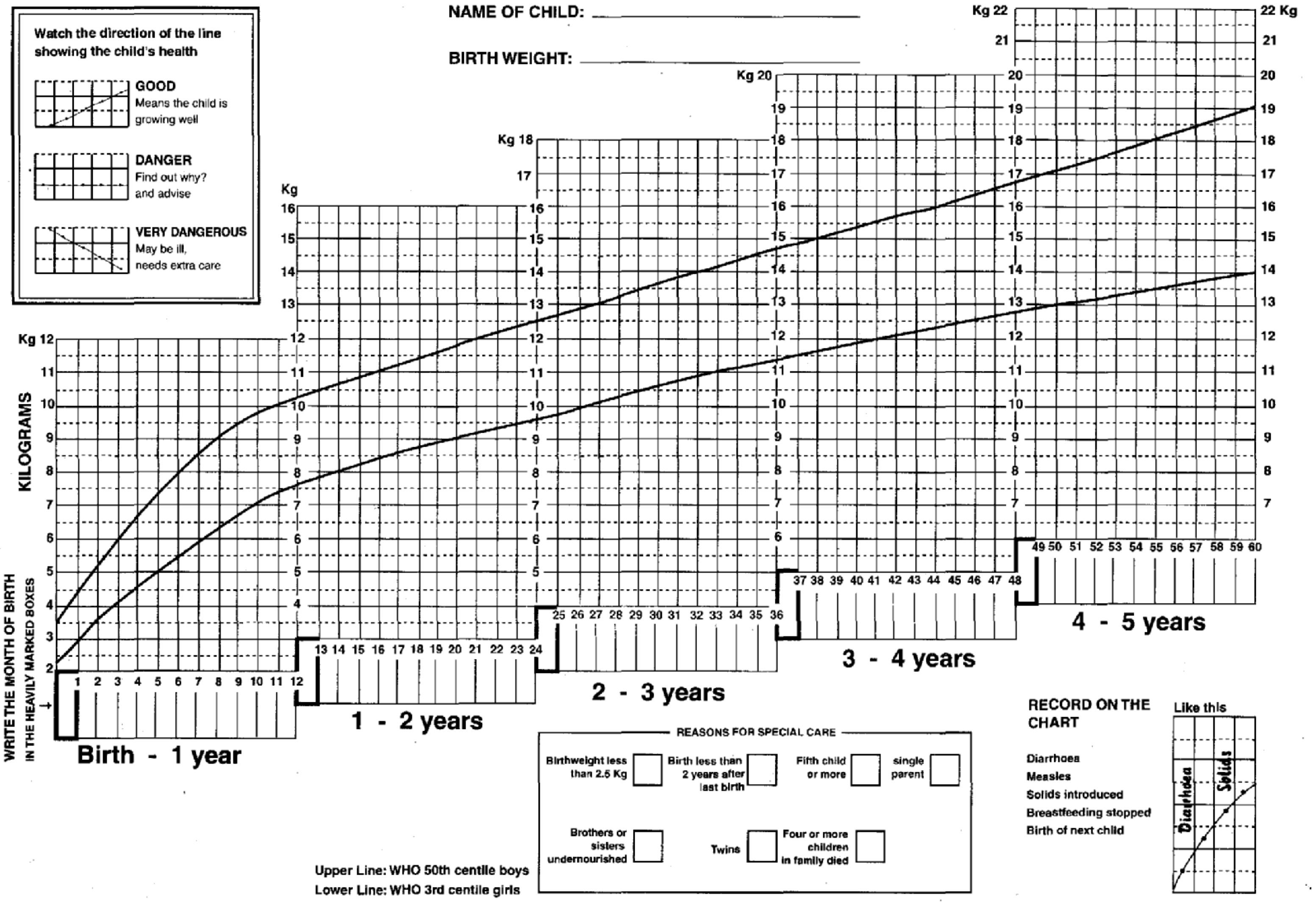

ANNEX IV Drugs with antimalarial activity that should not be used during the study

- chloroquine, amodiaquine;
- quinine, quinidine;
- mefloquine, halofantrine, lumefantrine;
- artemisinin and its derivatives (artemether, arteether, artesunate, dihydroartemisinin);
- proguanil, chlorproguanil, pyrimethamine;
- sulfadoxine, sulfalene, sulfamethoxazole, dapsone;
- primaquine;
- atovaquone;
- antibiotics: tetracycline*, doxycycline, erythromycin, azythromycin, clindamycin, rifampicin, trimethoprim;
- pentamidine

* Tetracycline eye ointments can be used.

ANNEX V Schedule of follow-up activities

	Days																		Months	
	0	1	2	3	4	5	7	8	9	10	11	12	14	15	16	28	35	42	2-11	12
**Medical history, previous medication, physical examination, consent**	X																			
**Pregnancy testing (if female age 13-49 years)**	X																			
**G6PD testing (Whatman 903)**		X																		
**Primaquine , 14d**			X	X	X	X	X	X	X	X	X	X	X	X	X					
**AL or CQ, 3d**	X	X	X																	
**Vital signs**	X	X	X	X			X						X			X	X	X	X	X
**Parasite microscopy**	X	X	X	X			X						X			X	X	X	X	X
**Hemoglobin**	X			X			X						X			X		X		X
**DBS for molecular testing (Whatman 903)**	X	X	(X)	(X)			(X)						(X)			(X)	(X)	(X)	(X)	(X)
**DBS for CQ drug level (Whatman 3 for CQ arms only (100µL) [40]**							(X)						(X)			(X)	(X)	(X)		
**DBS for lumefantrine level (Whatman 31Et Chr tartaric treated for AL arms only (100µL) [39]**							X													
**DBS for PQ drug level (Whatman 31Et Chr for PQ arms only (100µL)**							X						X							
**Adverse events monitoring**	X	X	X				X						X			X	X	X		
**History of fever in the previous 28 d**																			X	X
**Final visit**																				X

NOTES: Parentheses denote conditional or optional activities. The patient should be examined for parasitaemia if he or she has any danger signs. Rescue treatment could be given on any day, provided that the patient meets the criteria for treatment failure. Extra

days are any days other than regularly scheduled follow-up days when the patient returns to the facility because of recurrence of symptoms. On extra days, blood slides may be taken routinely or at the request of the clinical staff.

ANNEX VI Study Schedule

Day 0:

Screening

Clinical assessment including measurement of weight and height — referral in case of severe malaria/danger signs
Measurement of axillary temperature
Parasitological assessment
Informed consent

Enrollment

Treatment, first dose with either AL or CQ
Hemoglobin, filter paper for PCR
Pregnancy testing, if female age 13-49 years

Day 1:

Clinical assessment — referral in case of severe malaria/danger signs
Measurement of axillary temperature
Parasitological assessment
G6PD testing and randomization if G6PD normal
Treatment, second dose or alternative treatment in case of early treatment failure

Day 2:

Clinical assessment — referral in case of severe malaria/danger signs
Measurement of axillary temperature
Parasitological assessment
Treatment, third dose or alternative treatment in case of early treatment failure
Treatment, with primaquine if assigned to CQ+PQ or AL+PQ arms following a normal G6PD test result

Day 3, Day 7, Day 14, Day 21, Day 28, Day 35, and Day 42:

Clinical assessment — referral in case of severe malaria/danger signs
Measurement of axillary temperature
Parasitological assessment
Alternative treatment in case of treatment failure
Hemoglobin/hematocrit (Day 3, 7, Day 14, Day 28, Day 42), filter paper sampling for molecular testing on any smear positive days, CQ level testing in case of treatment

failure, lumefantrine level testing on Day 7, and primaquine level testing on Day 7 and 14
Treatment with primaquine continued Days 2-16 if assigned to CQ+PQ or AL+PQ arm under direct observation

Week 4 through 52:

weekly phone call
- asking about fever episodes since the last contact
- asking about migration

Month 2 through 12:

Monthly blood sampling for subclinical parasitemia
Check hemoglobin at the end of follow up period (day 365)

Any other day:

Clinical assessment — referral in case of severe malaria/danger signs
Measurement of axillary temperature
Parasitological assessment
Hemoglobin/hematocrit if indicated clinically
Alternative treatment in case of treatment failure
DBS if smear positive

ANNEX VII Consent/Assent Form

Informed consent form (for adult consent and parental permission for child six years and under), and assent form (for child 7-17 years), for enrolment for malaria in vivo efficacy study (Flesch-Kincaid Reading Level 8.9)

Contact Person:
Dr. Zenebe Melaku
Country Director, Columbia University-ICAP in Ethiopia,
Kirkos subcity, Woreda 04, Debre-zeit road, Building #20
PO Box 5566
Addis Ababa, Ethiopia
zy2115@columbia.edu
+251 11 4674475 (office)
+251 9 11 22 5347 (mobile)
+251 11 4674554 (Fax)

Participation Duration: 1 year
Anticipated Number of Subjects: 480

Purpose
The purpose of this research study is to find out how well the treatment for vivax malaria is working in Ethiopia. This will help us to treat vivax malaria better in the future. We are asking you to be part of this study because you have malaria. This study is supported by the Federal Ministry of Health (FMOH), United States Agency for International Development, ICAP at Columbia University, and Centers for Disease Control and Prevention.

Introduction
We invite you or your child to take part in this study. It is important that you understand that taking part in this activity is entirely your choice and you may or may not experience personal benefit as a result. You may stop taking part at any time without penalty or loss of any benefits you are entitled to. Information from this study might help others. The purpose of the study, the benefits, risks, discomforts and other information are discussed below. You will be told of any new information that is discovered during the course of this study, which may affect your willingness to continue to be a part of the study. Up to 480 persons may be enrolled in this study. You will be followed closely for 42 days and then monthly for one year.

Background
Malaria is a sickness caused by a very small germ that can get into the body when a mosquito bites you. It can cause fevers, headaches, body aches, and weakness. If it is not treated, it can make a person very sick. When malaria is treated with the right drugs, it can be cured. Sometimes the germ that causes malaria can change and these drugs don't work as well.

Procedures
Taking part in this study will last one year and will include twenty visits to the clinic.

If you or your child agree to be in this study a staff person will check you or your child. At all your visits, a small amount of blood will be taken from the tip of the finger (about 3 drops) after a needle prick. The blood will be checked for the malaria germ.

Each time we do a blood test for malaria, we will put a few drops of blood onto a piece of paper. This blood will be used to learn about the malaria germ that causes the illness. If the drug does not work as well as it should, some of this blood will be sent to a reference laboratory in Addis Ababa and the United States to check why the drug did not work. This blood sample will be destroyed once the tests are done and not stored.

Please be assured that if you decide that you or your child should not participate in this study, you or your child will be treated for malaria.

If you agree to participate or for your child to, you will get 1 or 2 of 3 drugs that are considered safe and standard treatments. You or your child will get either artemether-lumefantrine (Coartem), chloroquine alone, Coartem with primaquine or chloroquine with primaquine. What drug you receive will be decided by chance (like the flip of the coin). You will only be given primaquine after confirming a normal glucose-6-phosphate dehydrogenase status, a blood disorder that makes you more prone to adverse events from primaquine.

You or your child will need to come to the clinic to swallow the tablets every day for the first three days (up to day 16 if taking primaquine). We will watch you for about an hour after you take the medicine to make sure you do not vomit. You might also receive medications that you will have to take at home.

We will ask you or your child to return to the clinic 1, 2, 3, 7, 14, 21, 28, 35, and 42 days, and monthly thereafter after the treatment starts so we can check to make sure that the malaria has been cured and does not return. We will give you a visit card so you know what days to come or have your child come.

At every visit we will do a finger prick blood test for malaria. A test to check for malaria in the blood will be done today and at every visit. At each visit, a staff person will check you or your child and ask some questions. If you or your child does not come to clinic, someone from the clinic will come to your home to check on you.

We expect that these drugs will work well in most people. But if the malaria is not cured or if it comes back within 28 days, we will provide treatment with another drug called quinine. This is the standard treatment in Ethiopia for this situation. This treatment should cure malaria. We will still keep checking to make sure that the malaria is fully cured.

If you or your child has a severe case of malaria, this will lead to admission to the hospital.

There, strong drugs and other care will be given based on standards in Ethiopia.

Risks

There may be risks or discomforts if you take part in this study.

The drugs can cause an upset stomach, vomiting, diarrhea, headache, dizziness, mild skin rash, and itching. But these are mostly mild and soon go away. Primaquine can cause anemia especially in those with a certain blood disorder. You will be tested for this blood disorder and will not be given primaquine if found to have this deficiency.

Rarely, more severe side effects can occur. If you or your child has severe side effects, like persistent vomiting, severe rash, dark urine or hemoglobinuria, or dizziness and weakness with low blood pressure, we will stop the drug. We will also advise that you be treated with different malaria drugs in the future.

You or your child should come to the clinic right away if the sickness does not get better or it worsens and if there are any side effects from the drugs. You or your child can come back anytime even if not on a day you are scheduled to.

Make sure that you or your child come to clinic for appropriate care and possible transfer to a hospital if you have fever, convulsions, become very sleepy or cannot be woken up, have a lot of vomiting, are unable to drink or eat, have painful rash or mouth sores or red eyes, chest pain or difficult breathing or very fast breathing.

There are very little effects from the finger pricks like pain, bleeding, bruising, fainting, or infection. But these are very rare. We will clean the finger before taking blood and will use new needles to draw the blood each time.

There may be other risks of taking part in this study that we don't know about. If we learn about other risks, we will let you know what they are so that you can decide whether or not you or your child want to continue to be in the study.

Benefits

You may or may not get personal (direct) benefit from taking part in this study.

There are possible benefits of taking part in this study which follow:

You or your child will be closely followed for 42 days and then monthly for a year to see how well the drugs are working.

There will be someone here at the clinic every day. You may come for a visit at any time if you feel that you are ill, even on nights or weekends or in between visits.

This study will also help Ethiopia learn which drugs work best in this region. This may help you or someone you know in the future.

Alternate Procedures
If you decide that you do not want to take part or have your child be in this study, treatment for malaria will be provided.

Confidentiality
The results or information about you will be kept confidential to the extent allowed by law. Although every effort will be made to protect the privacy of the information about you or your child that is kept in the clinic, absolute privacy cannot be guaranteed. By signing this document you grant permission for information about you or your child obtained during the study to be made available to:

- The investigator, staff members, and other medical staff who may be evaluating the study.

- Staff from Columbia University and New York Presbyterian Hospital, the Center for Disease Control and Prevention, and the Ethiopian Ministry of Health, including the Institutional Review Board (IRB)/ ethical committees.

Injuries
Staff members will assist you in obtaining medical treatment, including emergency treatment, hospital care and follow-up care as needed. Any hospital stay which occurs during follow-up period will be paid for. Compensation for injury that results from participation in this study is not available.

You do not give up any of your legal rights by signing this consent form.

Compensation
You will receive 35 Ethiopian Birr for each visit to pay for your travel to the clinic. If you are the caretaker accompanying a child over 6 years, you will receive 50 Birr.

Additional Costs
n/a

Participation

Taking part in this study is your choice. You can decide not to take part in or stop being in the study at any time. Your choice will not affect the treatment you receive for malaria. Also, none of the treatment you receive will be affected. You may leave the study at any time. This will not affect your health care, and you will still receive malaria treatment for free.

If a staff member needs to take you out of the study for any reason, then we will not continue to follow you. If you are removed from the study before the treatment is complete, or if the

medicine did not make you better, then you will be referred to the clinic and treated with another treatment as noted in Ethiopia malaria recommendations.

Additional Information

Questions

If you have questions or concerns about taking part in this study, you may speak with one of the staff.

If you have any questions later about the study, please contact Dr. Zenebe Melaku at 09 11 22 5347 or Dr. Tesfay Abreha at 0911 06 2800.

If you would like to withdraw from the study, please contact Dr. Zenebe Melaku at 09 11 22 5347 or Dr. Tesfay Abreha at 0911 06 2800.

If you would like to speak with someone not directly involved with the study about your rights, please contact Mrs. Hiwot Solomon at 0910 10 0255 at the Ministry of Health.

Statement of Consent

By signing or placing my thumbprint below, I am saying that:

I have read this form, or it has been read to me; I have been able to ask questions about it, and my questions have been answered.

For adults: I understand that my or my child's participation is voluntary and that I can leave the study at any time without it affecting my care.

For children aged 7-17 years: I understand that my participation is voluntary and that I can leave the study at any time without it affecting my care. My decision to participate is supported by my parent/ guardian but not forced by him/her.

Read assent addendum below.

I agree to enter this study. I agree to report any unexpected or unusual symptoms.

I have received a copy of this form.

Signing this form does not waive any of my legal rights.

For adult subjects

Person Obtaining Consent
Print Name _____________________ Signature ________________ Date ________

Witness
Print Name _____________________ Signature ________________ Date ________

Subject
Print Name _____________________ Date _______
_
Thumbprint__________ Or Signature _________________________

For subjects 1-17 years old

Person Obtaining Consent
Print Name _____________________ Signature ________________ Date ________

Witness
Print Name _____________________ Signature ________________ Date ________

Subject
Print Name _____________________

Parent/Guardian
Print Name _____________________ Date ________
Thumbprint__________ Or Signature _________________________

Assent for Children 7-17 years of age (Flesch-Kincaid Reading Level 2.1)**: (please read to 7-17 year olds)**

Introduction
The Federal Ministry of Health (FMOH), United States Agency for International Development, International Center for AIDS Care and Treatment Programs (ICAP) at Columbia University, and Centers for Disease Control and Prevention invite you to be in this research study.

What is the purpose of the study?
You have vivax malaria. Malaria is a sickness that you can get when a mosquito bites you. The purpose of this study is to find out how well the drugs for vivax malaria are working in Ethiopia.

How long would you need me?
You will come to the clinic twenty times over the next year.

What do you want me to do if I decide to be in the study?
If you agree to be in this study, a staff person will check you. We will take blood from your finger and ask you some questions each time you come. You will be given a drug to treat vivax malaria. You will need to come to the clinic to swallow the tablets every day for the first three days (or 16 days if getting primaquine too) and may take some tablets at home.

Are there any risks to me if I decide to be in the study?
There may be things you don't like if you are in this study. The needle prick might hurt. The drugs can cause an upset stomach, headache, dizziness, or itching. But these go away soon.

If something more serious happens, your parent will bring you to the clinic where you will get treatment or be sent to the hospital.

Are there any benefits from being in the study?
You may or may not benefit from this study. You will not pay for anything during the study. There will be someone here to see you every day.

Who should I call if I have any questions?
If you have additional questions, ask your parent. They will know who you can call.

Do I have to be in this study?
Taking part in this study is your choice. You can decide not to take part in or stop being in the study at any time. Your choice will not affect your treatment for malaria.

Assent: I was told about the study. I asked questions. I had my questions answered. I want to be in the study.

For subjects 7-17 years old

Person Obtaining Consent
Print Name _____________________
Signature _________________ Date _______________________

Witness
Print Name _____________________
Signature _________________ Date _______________________

Subject
Print Name _____________________

ANNEX VIII Material Transfer Agreement

Ethiopian Science and Technology Commission, Health Department
National Health Research Ethics Review Committee
Address: Tel. 534945 P.O. Box 2490 Fax 251-1-524400
e-mail estc@ethionet.et Addis Ababa – Ethiopia

This Material Transfer Agreement (MTA) has been prepared for use by ICAP and U.S. Centers for Disease Control and Prevention (CDC) in all transfer of research material (samples, derivatives, and specimens) related to the protocol.
Ethiopia antimalarial *in vivo* efficacy study 2012: Evaluating the efficacy of artemether-lumefantrine alone compared to artemether-lumefantrine plus primaquine and chloroquine alone compared to chloroquine plus primaquine for P*lasmodium vivax* infection

Provider: Columbia University ICAP, Addis Ababa, Ethiopia
Recipient: U.S. Centers for Disease Control and Prevention (CDC)
1. Provider agrees to transfer to recipient's designated (Jimee Hwang) the following research materials (specimen).

- Day 7 filter paper collection for lumefantrine level, day of treatment failure filter paper collection for CQ drug level testing, and day 7 and 14 filter paper collection for primaquine drug levels
- Filter paper collection from day 0 and on any smear-positive days for molecular analyses

The research material will only be used for research purposes as described in the protocol by recipient's investigator in designated laboratory for the research project described below, under suitable containment conditions. This research material will not be used for commercial purposes such as screening, production or sale for which a commercialization license may be required. Recipient agrees to comply with all National and International guidelines rules and regulations applicable to the Research Project and the handling of the Research Material.

a) Are the Research materials of human origin?

Yes ☒ No ☐

b) If yes, are they collected according to the details in the protocol and in adherence to National Health Research Ethics Review Committee (NERC) recommendations and their approval.

Yes ☒ No ☐

2. This research material and its derivatives will be used by recipient's investigator solely in connection with the following research project ("Research Project") **Ethiopia anti-malarial *in vivo* efficacy study 2012: Evaluating the efficacy of artemether-lumefantrine alone compared to artemether-lumefantrine plus primaquine and chloroquine alone compared to chloroquine plus primaquine for P*lasmodium vivax* infection**
described with specificity as follows to determine lumefantrine level from day 7 filter papers, to determine chloroquine drug levels from day of treatment failure filter papers, to determine

primaquine levels from day 7 and 14, to perform genetic analysis from day 0 and day of treatment failure filter papers to differentiate the recurrent parasitemias as identical vs. different from the original infection and identify the presence of resistance drug markers, whole-genome wide sequencing, gametocyte molecular testing from any smear positive days, and from day 0 to perform genetic analysis for identification of genetic variants of G6PD deficiency from the enrolled study participants in the Ethiopia anti-malarial *in vivo* efficacy study 2012

3. In all presentations or written publications concerning the research project, recipient will seek agreement of provider and acknowledge provider's contribution of this research material unless requested otherwise.

4. This research material represents a significant contribution on the part of provider and is considered proprietary to provider. Recipient therefore agrees to retain control over this research Material and further agrees not to transfer the research Material to other people not under her/his direct supervision without advance written approval of provider. The research material will be disposed of as agreed upon per protocol at the end of the drug level testing and molecular analyses completion of the project.

5. The provider does not take any responsibility for loss, damage, wastage or spoilage of the research material during or after shipment to the address provided by the Recipient under conditions agreed to in the protocol of shipment of the samples. This Research Material is provided as a service to the research community. IT IS BEING SUPPLIED TO RECIPIANT WITH NO WARRANTIES, EXPRESS OR IMPLIED, INCLUDING ANY WARRANTY OF MERCHANTABILITY OR FITNESS FOR A PARTICULAR PURPOSE. Provider makes no representations that the use of the research material will not infringe any patent or proprietary right of third parties.

6. The recipient shall notify the provider in witting of any intention, improvement, modification discovery or development to the material or the information made by Recipient or parties, collaborating with Recipient, here in after referred to as "invention". Nothing in this agreement shall, however, be construed as conveying to the provider any rights under any patents or other intellectual property to such invention, other than as explicitly provided herein. At its option the provider shall be entitled to receive sample of any materials derived from the Materials for its own research and evaluation purposes only.

7. The under- signed provider and Recipient expressly certify and affirm that the contents of any statements made herein are truthful and accurate.

8. Any additional terms (use an attached page if necessary):

9. The provider maintains, ownership right of the research material and its derivatives unless stated otherwise. The provider will retain a copy (aliquot) of every sample sent abroad as much as possible for local research needs.

Material Transfer Agreement Signature page

For Recipient:

Recipient's Investigator
Jimee Hwang
Signature

Date ------------------------
Mailing Address for Material:

Tel: --
Fax: ---
-

Duly Authorized
Venkatachalam Udhayakumar
Signature/ Stamp

Date ------------------------------
Mailing Address for Notices:

Tel: ---
Fax: --

For Provider

Provider's Investigator
Tesfay Abreha
Signature

Date ------------------------
Mailing Address for Material:

P.O. Box --
-
Tel: --
-
Fax: ---
-

Duly Authorized
Zenebe Melaku
Signature/ Stamp

Date ------------------------------
Mailing Address for Notices:

P.O. Box ---
Tel: --
Fax: --
