# Supplementary material for: Comparison of artemether-lumefantrine and chloroquine with and without primaquine for the treatment of Plasmodium vivax infection in Ethiopia: A randomized controlled trial
Source: PLoS Med. 2017 May 16;14(5):e1002299. doi: 10.1371/journal.pmed.1002299 (PMC5433686; doi:10.1371/journal.pmed.1002299)
Supplement: S2 Text — (PDF) [file pmed.1002299.s005.pdf]

Genotyping

Molecular processing:

PCR-based species confirmation was undertaken on all day 0 and day of failure clinical samples using the previously described Genus/*P. falciparum* duplex PET-PCR assay [1] and an in-house *P. vivax* PET-PCR assay. Parasite genotyping was undertaken using seven microsatellite markers described previously [2, 3] using a commercial PCR MasterMix (Promega, Madison, WI). Length variation in fluorescently-labelled PCR products was determined on an Applied Biosystems Prism 3130xl Avant Genetic Analyzer and the data analyzed using GeneMapper v4.0 (all Applied Biosystems, Foster City, CA). Paired samples with the same microsatellite profile were considered homologous recurrences, while profiles with at least one microsatellite difference among the seven used were classified as heterologous recurrences.

Analysis of PCR adjustment

Parasite genotyping can determine whether a recurrent parasitaemia is homologous to the initial infection. Recurrence of *P. vivax* genetically identical to the pre-treatment isolate can occur from either a true recrudescence of the initial infection or a relapse from hypnozoites generated from the prior blood stage infection; current molecular methods are unable to distinguish between these alternatives. However PCR adjusted efficacy can reduce the confounding effects of recurrent parasitaemias arising from new infection or relapse from a different strain. For this purpose recurrent samples are defined as homologous (recrudescence/relapse) if at least one allele is shared with the day 0 sample at each locus investigated and heterologous (re-infection/relapse) if no alleles are shared with the day 0 samples at 1 or more loci. Recurrence outcomes are defined as indeterminate if a sample pair exhibits no informative data at all loci investigated. Informative data from a minimum of 3 loci is required to call a homologous recurrence event and one locus to call a heterologous event. For calculation of the PCR-adjusted cure rate, homologous recurrences are classified as recrudescences (although these may include homologous relapses) and heterologous recurrences are classified as re-infections or heterologous relapses and are being censored

Assessment of heterozygosity

The capacity of the genotyping markers to distinguish heterologous from homologous infections in the study population was assessed by measuring *P*, the probability that two unrelated parasites would exhibit homologous genotypes (i.e. expected homozygosity), using the day 0 (baseline) samples[4]. *P* was calculated using the following formula: $P = [n/(n\text{-}1)][\Sigma p^2 i]$, where *n* is the number of isolates analyzed and *pi* is the frequency of the *ith* allele in the population. Cumulative probabilities (πP_i) of two unrelated parasites exhibiting homologous genotypes at multiple loci were calculated by multiplying *P* for the individual loci in question. As summarised in the table below, the cumulative probability of two unrelated parasites exhibiting homologues genotypes across all 7 loci was 3.9×10^{-5}. Expected heterozygosity values (H_E) are also provided for each locus for comparisons with other studies. H_E is related to *P*, providing a measure of the probability that two unrelated parasites will exhibit different genotypes at a given locus using the formula: $H_E = [n/(n\text{-}1)][1\text{-}\Sigma p^2 i]$. The average H_E in the study population was 0.75, reflecting moderately high diversity. Similar levels of diversity have been observed in previous studies of *P. vivax* in Ethiopia [5, 6].

Table A: Assessment of heterozygosity

Locus	Allelic size range, bp	Expected Heterozygosity (H_E)	Cumulative *P* (πP_i)
MS6	233-263	0.84	1.6×10^{-1}
Pvms007	345-441	0.79	3.4×10^{-2}
MS2	180-266	0.78	7.4×10^{-3}
Pv3502	124-281	0.78	1.6×10^{-3}
Pvms038	186-236	0.78	3.6×10^{-4}
Pvms008	230-369	0.75	9.0×10^{-5}
Pv1162	176-221	0.56	3.9×10^{-5}

PCR Adjusted results:

Genotyping was feasible in 362 (90.9%) parasite isolates on day 0 and 90.2% (46/51) of paired isolates from patients with recurrence prior to day 42. In total 22 (47.8%) of the 46 paired parasite isolates that could be assessed were homologous: 7 (41.2%) in the CQ arm, 11 (50%) in the AL arm and 4 (66.7%) in the AL+PQ arm.

Tables B: Cumulative risk of recurrence and hazard ratios from survival analyses after censoring heterologous infections

		Chloroquine	Chloroquine Plus Primaquine	Artemether-Lumefantrine	Artemether-Lumefantrine plus Primaquine
		**N=104**	**N=102**	**N=100**	**N=92**
**Day 28**	**Adjusted cumulative risk of *P. vivax* (% [95%CI])**	2.1 [0.5 – 8.3]	0	7.0 [3.2- 15.0]	2.5 [0.6- 9.5]
**Day 42**	**Adjusted at cumulative risk of *P. vivax* (% [95%CI])**	7.9 [3.8- 15.8]	0	17.5 [10.7 – 27.8]	3.7 [1.2 – 11.1]

		Chloroquine Vs Chloroquine Plus Primaquine[a]	Artemether-Lumefantrine Vs Artemether-lumefantrine plus primaquine[a]	Artemether-lumefantrine vs Chloroquine	Artemether-lumefantrine plus primaquine vs Chloroquine plus primaquine
**Day 28**	**PCR Adjusted Hazard Ratio**	- p=0.164	2.6 [0.1-2.0] p=0.257	3.2 [0.7-16.0] p=0.150	- p=0.139
**Day 42**	**PCR Adjusted Hazard Ratio**	- p=0.0008	4.9 [1.4-17.2] p=0.013	2.4 [1.0-6.1 -] p=0.053	- p=0.068

Hazards Ratio [95% Confidence Intervals] calculated from Cox regression. [a]excluding patients not randomized to PQ vs non-PQ

References

1. Lucchi NW, Karell MA, Journel I, Rogier E, Goldman I, Ljolje D, et al. PET-PCR method for the molecular detection of malaria parasites in a national malaria surveillance study in Haiti, 2011. Malaria journal. 2014;13:462. doi: 10.1186/1475-2875-13-462. PubMed PMID: 25428550; PubMed Central PMCID: PMC4289323.
2. Imwong M, Nair S, Pukrittayakamee S, Sudimack D, Williams JT, Mayxay M, et al. Contrasting genetic structure in Plasmodium vivax populations from Asia and South America. International journal for parasitology. 2007;37(8-9):1013-22. doi: 10.1016/j.ijpara.2007.02.010. PubMed PMID: 17442318.
3. Joy DA, Gonzalez-Ceron L, Carlton JM, Gueye A, Fay M, McCutchan TF, et al. Local adaptation and vector-mediated population structure in Plasmodium vivax malaria. Molecular biology and evolution. 2008;25(6):1245-52. doi: 10.1093/molbev/msn073. PubMed PMID: 18385220; PubMed Central PMCID: PMC2386084.
4. Gatton ML, Cheng Q. Can estimates of antimalarial efficacy from field studies be improved? Trends in parasitology. 2008;24(2):68-73. doi: 10.1016/j.pt.2007.11.003. PubMed PMID: 18182325; PubMed Central PMCID: PMC2646162.
5. Hwang J, Alemayehu BH, Reithinger R, Tekleyohannes SG, Takele T, Birhanu SG, et al. In vivo efficacy of artemether-lumefantrine and chloroquine against Plasmodium vivax: a randomized open label trial in central Ethiopia. PloS one. 2013;8(5):e63433. doi: 10.1371/journal.pone.0063433. PubMed PMID: 23717423; PubMed Central PMCID: PMC3661577.
6. Getachew S, To S, Trimarsanto H, Thriemer K, Clark TG, Petros B, et al. Variation in Complexity of Infection and Transmission Stability between Neighbouring Populations of Plasmodium vivax in Southern Ethiopia. PloS one. 2015;10(10):e0140780. doi: 10.1371/journal.pone.0140780. PubMed PMID: 26468643; PubMed Central PMCID: PMC4607408.
