# Supplementary material for: Comparison of artemether-lumefantrine and chloroquine with and without primaquine for the treatment of Plasmodium vivax infection in Ethiopia: A randomized controlled trial
Source: PLoS Med. 2017 May 16;14(5):e1002299. doi: 10.1371/journal.pmed.1002299 (PMC5433686; doi:10.1371/journal.pmed.1002299)
Supplement: S3 Text — (PDF) [file pmed.1002299.s006.pdf]

Statistical Analysis Plan (SAP)

Comparison of artemether-lumefantrine and chloroquine with and without primaquine for the treatment of *Plasmodium vivax* in Ethiopia: a randomized controlled trial

Contents SAP

1 Introduction 3
2 Study design and objectives 3
2.1 Study design 3
2.2 Sample size 4
2.3 Study objectives 4
3 Endpoints 5
3.1 Demographic and baseline characteristics 5
3.2 Efficacy endpoint 5
3.2.1 Primary efficacy endpoints 5
3.2.2 Secondary efficacy endpoints 5
3.3 Safety endpoints 6
3.4 Endpoints additional analyses: drug levels 6
4 Definitions and outcome adjudications 6
4.1 Definition of treatment outcomes at day 28 and day 42 6
4.1.1 Early treatment failure 6
4.1.2 Late treatment failure 6
4.1.3 Adequate clinical and parasitological response 7
4.2 Adjudication of day 28 and 42 (Phase 1) treatment outcome assessment 7
4.2.1 Missing Blood smears: 7
4.2.2 PCR adjustment: 7
4.3 Adjudication of month 12 (Phase 2) treatment outcome assessment 8
4.4 Adjudication for endpoint on parasite prevalence on day 0-3 8
4.5 Adjudication for endpoint on fever clearance on day 0-3 8
4.6 Adjudication of hemoglobin outcomes 8
4.7 Handling of missing data on drug course 9
4.8 Handling of missing data for Adverse Events 9

5 Analyses population 9
5.1 Efficacy population 9
5.1.1 Intention to treat 9
5.1.2 modified Intention to Treat 10
5.2 Safety Population 10
5.3 Additional analyses (CQ drug concentrations) population 10
6 Statistical methods 10
6.1 Demographic and Baseline 10
6.1.1 Demographic characteristics 10
6.1.2 Disease characteristics at baseline 10
6.1.3 Prior or concomitant medications 10
6.2 Efficacy analyses 11
6.3 Analyses of primary efficacy endpoints 11
6.3.1 Cumulative risk of *P. vivax* treatment failure at day 28/42 following treatment with AL compared to AL+PQ (Qu. A) 11
6.4 Analyses of secondary efficacy endpoints 11
6.4.1 Schizontocidal Endpoints: 11
6.4.2 Radical Cure Endpoints: 12
6.4.3 Effectiveness endpoints 13
6.5 Analyses of safety data 13
6.5.1 Proportion of patients completing 3 days of schizontocidal treatment 14
6.5.2 Proportion of patients completing 14 days of primaquine treatment in the two primaquine treatment arms 14
6.5.3 Dose Vomiting 14
6.5.4 Analysis of Adverse Events and Severe Adverse Events 14
6.5.5 Haematological Recovery 14
6.6 Analyses of pharmacokinetic data (drug concentrations) 14
6.6.1 CQ concentration at day of recurrence 14

1 Introduction

This Statistical Analysis Plan (SAP) provides a detailed and comprehensive description for the analysis of the antimalarial efficacy study entitled "Evaluating the efficacy of artemether-lumefantrine alone compared to artemether-lumefantrine plus primaquine and chloroquine alone compared to chloroquine plus primaquine for *Plasmodium vivax* infection". The purpose of the study is to assess the therapeutic efficacy of arthemether-Lumefantrine (AL) compared to artemether-lumefantrine + primaquine (AL+PQ) and chloroquine (CQ) compared to chloroquine + primaquine (CQ+PQ) for *P. vivax* infection and to determine the number of recurrent vivax episodes in patients receiving radical cure compared to those who did not . The study design is described in a Protocol titled "Ethiopia antimalarial in vivo efficacy study 2012: Evaluating the efficacy of artemether-lumefantrine alone compared to artemether-lumefantrine plus primaquine and chloroquine alone compared to chloroquine plus primaquine for Plasmodium vivax infection" and the trial registered with ClinicalTrials.gov (NCT01680406).

The analysis will be presented in a report, which will be used as the basis of the primary research publications according to the study publication plan. This SAP describes the statistical methods for the primary and secondary outcomes of the study as defined in the protocol as well as additional pharmacokinetic analyses.

2 Study design and objectives

2.1 Study design

The aim of the study is to improve the radical cure of *P. vivax*. This requires the elimination of blood stage parasites with schizontocidal drugs, followed by the removal of the dormant liver stages with a hypnozonticidal agent; the trial design aims to define these two antimalarial modes of action.

The trial is a randomised, open label evaluation of clinical and parasitological responses to directly observed treatment for uncomplicated *P. vivax* malaria. Patients with uncomplicated *P. vivax* mono-infection who meet the study inclusion criteria are enrolled, randomised, and treated with either AL or CQ with or without PQ, and monitored for 12 months. Patients with recurrent episodes of malaria are treated again with the same regimen and continue to be followed. The first treatment episode is partially supervised while subsequent treatment with primaquine is unsupervised. Patients failing in any arm within 14 days of treatment are treated with rescue treatment (Quinine for 7 days). Patients presenting with vivax parasitaemia > 14 days following treatment will receive the same radical therapy as at enrollment.

The recurrence of *P. vivax* parasitaemia during follow up can result from either recrudescence of the same parasite, reinfection with a new parasite, or relapse from a liver stage parasite which can be the same or different from the initially observed parasite[1,2].

[1] White, N.J., *The assessment of antimalarial drug efficacy.* Trends Parasitol, 2002. **18**(10): p. 458-64.
[2] Price, R.N., et al., *Phenotypic and genotypic characterisation of drug-resistant Plasmodium vivax.* Trends Parasitol, 2012. **28**(11): p. 522-9.

Failure of schizontocidal treatment (CQ and AL) can result in recrudescence of the same parasite early during follow up (usually before 42 days), whereas failure from antirelapse treatment can occur from approximately 16 days to over a year. The earliest time of recurrence is dependent upon the pharmacokinetic profile of the antimalarial treatment regimen which defines the post treatment prophylaxis.

2.2 Sample size

The design of the clinical study is to compare four treatment arms AL or CQ alone with or without primaquine. From a previous efficacy study, CQ monotherapy had an efficacy of 68% at day 42 (null hypothesis). It is assumed that the addition of PQ will decrease the chance of relapse by at least 60% resulting in a success in 87% of patients. Group sample sizes of 97 in each group were set to achieve 90% power to detect a difference between group proportions of 19% using the two-sided Z-test with pooled variance test statistic. The significance level of the test was targeted at 0.05. Adjusting the alpha level to 0.025 for multiple comparisons will increase the sample size to 118 in each group. The AL arm showed even a lower efficacy at day 42 in our previous study of 58%.

Using this lower success rate of 58% seen with AL at day 42, resulted in a smaller required sample size of 68. Taking into account 10-20% loss to follow-up at 42 days, a sample size of 120 subjects for each treatment was determined to be optimal.

2.3 Study objectives

The primary objective is to assess the day 28/42 therapeutic efficacy of AL compared to AL + PQ and CQ compared to CQ + PQ against *P. vivax* infection. The secondary objective to compare AL vs CQ and AL+PQ vs CQ+PQ and also the overall number of

**Study Question**	**Endpoints for Comparison**
A) Does the addition of 14 day course of supervised primaquine reduce the risk of treatment failure by 28/42 days	Comparison of efficacy of CQ versus CQ+PQ and AL versus AL+PQ at day 28/42. First episode of malaria only.
B) What is the most efficacious schizontocidal treatment for *P. vivax*?	Comparison of CQ versus AL efficacy at day 28 and day 42. First episode of malaria only.
C) What is the most effective treatment for early parasite clearance?	Comparison of parasitaemia day 0-3 and fever clearance CQ vs CQ+PQ and AL vs AL+PQ and AL vs CQ
D) What is the efficacy of low dose primaquine for radical cure?	Comparison of the cumulative risk and incidence rate of *P. vivax* recurrences at 12 months for CQ vs CQ+PQ and AL vs AL+PQ.
E) What is the most efficacious radical cure for *P. vivax*?	Comparison of the cumulative risk of recurrence and incidence rate of *P. vivax* at 12 months for CQ+PQ vs AL+PQ
F) What is the effectiveness of unsupervised primaquine	Comparison of the cumulative risk and incidence rate of *P. vivax* recurrences at 12 months for CQ vs CQ+PQ and AL vs AL+PQ, using the second episodes of malaria (unsupervised) as starting point for the analyses

Phase 1 of the study evaluates the clinical, parasitological, and haematological parameters for *P. vivax* infection over a 42-day follow-up period, to compare the schizontocidal drug efficacy of CQ

and AL. Phase 2 continues the monthly follow-up of these patients for one year to assess the frequency of recurring vivax infections and thus the overall radical cure of CQ+PQ and AL+PQ over 12 months. The comparison of each drug (CQ and AL) with and without primaquine defines the antirelapse efficacy of the currently recommended dose of primaquine for *P. vivax* in Ethiopia.

3 Endpoints

3.1 Demographic and baseline characteristics

Once enrolled, all patients are randomized into four treatment groups. Patients in each group will be described with respect to baseline characteristics. These will include means (standard deviations) or medians (interquartile ranges) for non-normally distributed data. Categorical characteristics will be presented as counts and percentages.

3.2 Efficacy endpoint

3.2.1 Primary efficacy endpoints

- The cumulative risk (time to first event) of *P. vivax* treatment failure at day 28/42 following treatment of the first episode of malaria, comparing AL with AL+PQ (Qu. A)
- The cumulative risk (time to first event) of *P. vivax* treatment failure at day 28/42 following treatment of the first episode of malaria, comparing CQ with CQ+PQ (Qu. A)

3.2.2 Secondary efficacy endpoints

3.2.2.1 Schizontocidal Endpoints:

- The cumulative risk (time to first event) of *P. vivax* treatment failure at day 28 and 42 following treatment of the first episode of malaria, comparing AL and CQ (Qu. B)
- PCR adjusted cumulative risk (time to first event) of *P. vivax* at day 28 and 42 following treatment of the first episode of malaria comparing AL with CQ (Qu. B)
- cumulative risk of any parasitaemia (all species) at day 28 and 42 following treatment, comparing AL with CQ (Qu. B)
- Proportion of patients with *P. vivax* parasitaemia on day 1, 2 and 3, comparing AL with CQ, AL with AL+PQ, and CQ with CQ+PQ(Qu. C)
- Proportion of patients with fever on day 1, 2 and 3 after treatment , comparing AL with CQ, AL with AL+PQ, and CQ with CQ+PQ(Qu. C)

3.2.2.2 Radical Cure Endpoints:

- Incidence rate of all recurrent episodes of *P. vivax* parasitaemia over one year, comparing CQ with CQ+PQ, AL with AL+PQ and CQ+PQ with AL+PQ (Qu. D & E)
- The cumulative risk (time to first event) of recurrent *P. vivax* parasitaemia over one year following treatment, comparing CQ with CQ+PQ, AL with AL+PQ and CQ+PQ with AL+PQ (Qu. D & E)
- The incidence rate of any parasitaemia at 1 year following treatment (includes any species of infection), comparing CQ with CQ+PQ, AL with AL+PQ and CQ+PQ with AL+PQ (Qu. D &E)

3.2.2.3 "Effectiveness" endpoints:

- The cumulative risk (time to first event) of recurrent *P. vivax* parasitaemia at 6 months following treatment of the second episode of malaria (unsupervised treatment), comparing with the enrollment episode (Qu. F).

3.3 Safety endpoints

- Proportion of patients completing 3 days of schizontocidal treatment
- Proportion of patients completing 14 days of partially supervised primaquine treatment in the two primaquine treatment arms
- Proportion of treatment days on which patients vomited drugs within 1 hour of administration
- Proportion of patients with adverse and serious adverse events
- Fractional change in Hb between baseline and day 3, 7 and again on day 28
- Proportion of patients with >25% drop in Hb from baseline within 7 days
- Proportion of patients with anaemia less than 7g/dl on day 3 and day 7

3.4 Endpoints additional analyses: drug levels

- CQ concentration at day of recurrence

4 Definitions and outcome adjudications

4.1 Definition of treatment outcomes at day 28 and day 42

Treatment outcomes at day 28 and 42 will be divided into early treatment failure, late treatment failure and adequate clinical and parasitological response[3].

4.1.1 Early treatment failure

- Danger signs or severe malaria on day 1, 2 or 3 in the presence of peripheral parasitaemia
- Parasitaemia on day 2 higher than on day 0, irrespective of axillary temperature
- Parasitaemia on day 3 with axillary temperature ≥ 37.5 ºC
- Parasitaemia on day 3 ≥ 25% of count on day 0

4.1.2 Late treatment failure

4.1.2.1 Late clinical failure

- Danger signs or severe malaria in the presence of parasitaemia on any day between day 4 and day 28 (day 42) in patients who did not previously meet any of the criteria of early treatment failure;
- Presence of parasitaemia on any day between day 4 and day 28 (day 42) with axillary temperature ≥ 37.5 ºC (or history of fever) in patients who did not previously meet any of the criteria of early treatment failure

[3] WHO, *Methods for Surveillance of Antimalarial Drug Efficacy*, 2009.

4.1.2.2 Late parasitological failure

Presence of parasitaemia on any day between day 7 and day 28 (day 42) with axillary temperature < 37.5 ºC in patients who did not previously meet any of the criteria of early treatment failure or late clinical failure

4.1.3 Adequate clinical and parasitological response

Absence of parasitaemia on day 28 (day 42), irrespective of axillary temperature, in patients who did not previously meet any of the criteria of early treatment failure, late clinical failure or late parasitological failure. Adjudication of efficacy endpoints at day 28 and day 42.

4.2 Adjudication of day 28 and 42 (Phase 1) treatment outcome assessment

4.2.1 Missing Blood smears:

The clinical definition of TF enables determination of the primary efficacy endpoint for most patients. However when the number of scheduled visits is incomplete the following rules will be applied[4]:

Deviation	Effect
More than 18 days without blood smear results	Lost to follow up on day of last visit
No blood smear results between D25 and D31	Lost to follow up on day of last visit
No blood smear results between D39 and D45	Lost to follow up on day of last visit

Detection of *non-vivax* parasitaemia before day 28 or 42 of follow up will result in readmission of antimalarial treatment, and thus these patients will be censored for the schizontocidal efficacy analyses (Qu A and B).

4.2.2 PCR adjustment:

Parasite genotyping can determine whether a recurrent parasitaemia is homologous to the initial infection. Recurrence of *P. vivax* genetically identical to the pre-treatment isolate can occur from either a true recrudescence of the initial infection or a relapse from hypnozoites generated from the prior blood stage infection; current molecular methods are unable to distinguish between these alternatives. However PCR adjusted efficacy can reduce the confounding effects of recurrent parasitaemias arising from new infection or relapse from a different strain. For this purpose recurrent samples are defined as homologous (recrudescence/relapse) if at least one allele is shared with the day 0 sample at each locus investigated and heterologous (re-infection/relapse) if no alleles are shared with the day 0 samples at 1 or more loci. Recurrence outcomes are defined as indeterminate if a sample pair exhibits no informative data at all loci investigated. Informative data from a minimum of 3 loci is required to call a homologous recurrence event and one locus to call a heterologous event. For calculation of the PCR-adjusted cure rate, homologous recurrences are classified as recrudescences (although these may include homologous relapses) and heterologous recurrences are classified as re-infections or heterologous relapses and are being censored. .

Patient data with samples negative for *P. vivax* by PCR at enrollment will be excluded in this analyses.

---

[4] WWARN. *Clinical Module, Data Management and Statistical Analyses Plan*. 2013; Available from: http://www.wwarn.org/sites/default/files/ClinicalDMSAP.pdf

4.3 Adjudication of month 12 (Phase 2) treatment outcome assessment

When the number of scheduled visits after day 42 is incomplete the following rules will be applied:

Deviation	Effect
More than 45 days without blood film examination after day 42	-In the survival analysis lost to follow up on day of last visit<br>-In the incidence rate analysis the period of missing observation will be deducted from the total period of observation.

4.4 Adjudication for endpoint on parasite prevalence on day 0-3

For missed blood films (microscopy) the following rules will be applied:

Parasitaemia				
Day 0	Day 1	Day 2	Day 3	**Effect**
Yes	Yes	No	Missing	Assumed no parasitaemia on day 3
Yes	Yes	Missing	Yes	Assumed parasitaemia on day 2
Yes	No	Missing	Missing	Assumed no parasitaemia on day 2 and 3
Yes	Yes	Missing	No	Will not contribute to proportion of patients with parasitaemia on day 2
Yes	No	Missing	No	Assumed no parasitaemia on day 2
Yes	No	Missing	Yes	Will not contribute to proportion of day 2 with parasitaemia
Yes	Yes	Missing	Missing	Will not contribute to proportion of patients with parasitaemia on day 2 or 3

4.5 Adjudication for endpoint on fever clearance on day 0-3

For missed temperature records, the same rules as for missing blood slides will apply.

4.6 Adjudication of hemoglobin outcomes

For missing haemoglobin measurements during follow up the following rules apply:

Outcome assessment	Deviation	Action
Fractional change in Hb between baseline and day 3	Missing Hb measurement on day 3	Exclude from analysis
Fractional change in Hb between baseline and day 7	Missing Hb measurement on day 7	Exclude from analysis
Fractional change in Hb between baseline and day 28	Missing Hb measurement on day 28	Exclude from analysis

Proportion of patients with >25% drop in Hb from baseline within 7 days	Missing Hb measurement on day 7	Exclude from analysis
Proportion of patients with anaemia less than 7g/dl on day 3	Missing day 3 Hb measurement	Exclude from analysis
Proportion of patients with anaemia less than 7g/dl on day 7	Missing day 7 Hb measurement	Exclude from analysis

4.7 Handling of missing data on drug course

Patients can have an incomplete course of treatment or data on drug administration may be missing. No imputation of treatment course will be made for patients with missing data. Subgroup analyses may be performed for patients with full versus partial treatment courses if appropriate.

4.8 Handling of missing data for Adverse Events

For patients with missing data on adverse events the most conservative approach will be used:

**Deviation**	**Action**
Start date of AE missing	Assume during study drug intake
End date of AE missing	No imputation
Date of start of study treatment administration missing	All AEs after randomization considered to have happened during study drug intake
Missing assessment of relationship to study treatment	Assume event to be possibly related
Missing severity assessment of AE	Assume highest severity

5 Analyses population

5.1 Efficacy population

For the efficacy analysis, an intention-to-treat (ITT) and modified Intention to treat (mITT) approach will be adopted, with the ITT analysis being the primary approach, to provide comparison of the different drug treatments.

5.1.1 Intention to treat

To provide a pragmatic comparison of the different drug treatments, the principle of intention-to-treat, will be the main strategy of analysis adopted for the primary and secondary endpoints. These analyses will be conducted on all patients assigned to the treatment groups as randomized, regardless of the study treatment received.

5.1.2 modified Intention to Treat

A modified intention to treat approach will be used as secondary analyses. Patients with protocol violations (e.g. wrong treatment or no treatment information available) will be censored form the respective episode onwards.

5.2 Safety Population

For the analysis of safety outcomes, all patients who effectively received any drug (i.e. at least one treatment dose) are included in the safety analysis in the treatment group they actually received.

5.3 Additional analyses (CQ drug concentrations) population

For the analyses of CQ drug levels only patients who failed before day 28 in the CQ arm and who had blood collected for drug levels will be included.

6 Statistical methods

6.1 Demographic and Baseline

Details of patients screened, those who meet the study inclusion criteria, those who are eligible and randomized, those who are eligible but not randomized (e.g. G6PD deficient), those who withdraw from the study after randomization and those who are lost to follow-up will be summarized in CONSORT flow diagrams (one until day 42 endpoint and one until end of study).

The number of patients discontinuing from the study will be tabulated by reason for study discontinuation. The number (%) of patients attending scheduled follow-up visits by study day (days 1 to 28 and 42, monthly visits until 1 year) will be reported.

The baseline value is defined as the last available value before randomization.

6.1.1 Demographic characteristics

- Gender: male / female
- Median Age (years)
- Age in classes: 12 months up to 5 years, 5 years up to 15 years, ≥15 years
- Median Weight

6.1.2 Disease characteristics at baseline

Specific disease history including the parasite density (/µL) and Gametocyte density (/µL) at D0, as well as axillary temperature (°C) (quantitative and if <37.5°C) and signs and symptoms (weakness, headache, anorexia, nausea, vomiting, pain, diarrhea, convulsion, dehydration, icterus, sweat, chills, skin disorders) & pulse rate (beats/min) at D0.

6.1.3 Prior or concomitant medications

All medications taken within fourteen days before randomization and until the end of the study are reported in the case report form pages.

- Prior medications are those the patient used prior (seven days before) to first study drug intake. Prior medications can be discontinued before first administration or can be ongoing during treatment phase.
- Concomitant medications are those used during the treatment with the study drug and

during the first phase of follow up until day 42. Concomitant medications can be ongoing prior medication and/or continue into post treatment medication.

- Post treatment medications are those the patient took in the period running from the day after day 42 up to the end of the study.

6.2 Efficacy analyses

In this study, patients with repeated episodes are treated with the same regimen they received for their initial episode. However only the first episode is mostly directly observed (all schizonticidal treatment is observed but primaquine administration is partially observed), whereas for all consecutive episodes patients are advised to take their treatment at home (unsupervised). The analyses for schizontocidal efficacy therefore only takes the first episode into consideration (enrollment episode). For the “**effectiveness” analyses** only episodes following the first recurrences (ie second and greater episodes, using the second episode as starting point) will be included.

6.3 Analyses of primary efficacy endpoints

6.3.1 Cumulative risk of *P. vivax* treatment failure at day 28/42 following treatment with AL compared to AL+PQ (Qu. A)

The response to treatment will be assessed using survival analyses (Kaplan Meier). Comparisons between treatment arms will be presented as Hazard Ratios using a Cox regression model. The proportional hazards assumption will be assessed by comparing visually the log(cumulative hazard) by time of follow-up curves for each covariable category and subsequently by fitting and comparing models with and without time of follow-up interaction terms.

In the survival analyses patients with a recurrent vivax parasitaemia before day 28/42 are considered failures, patients lost to follow up or with other a non-vivax parasitaemia before day 28/42 are censored at the respective day. Patients with no recurrence until day 28/42 are considered cured for the purpose of this analysis. Incidence risk of *P. vivax* treatment failures at day 28/42 following treatment with CQ compared to CQ+PQ (Qu. A)

6.4 Analyses of secondary efficacy endpoints

6.4.1 Schizontocidal Endpoints:

All analyses for schizontocidal endpoints refer to the first malaria episode (enrollment episode).

6.4.1.1 Cumulative risk of *P. vivax* treatment failures at day 28 and day 42 following treatment with CQ compared to AL (Qu. B)

The incidence risk of recurrent *P. vivax* over 28 and 42 days will be assessed using survival analyses (Kaplan Meier), similar to 6.3.1.

6.4.1.2 PCR-adjusted cumulative risk of *P. vivax* at day 28 and 42 following treatment with AL compared to CQ (Qu. B)

The incidence risk (cumulative risk) of recurrent *P. vivax* over 28 and 42 days will be assessed by survival analysis (Kaplan Meier), similar to 6.3.1, but parasites with different genotypes will be censored from the analysis. If in all of the markers used at least one allele is matching in the enrollment sample and in the sample taken at the day of recurrence, the recurrence will be considered the same infection. Otherwise, it will be considered a different infection. In the event

that molecular data is missing from the day of recurrence, the patient will be censored at this day without a recorded endpoint outcome (i.e., that patient will not contribute any influence on the risk estimate).

6.4.1.3 Cumulative risk of any parasitaemia at day 28 and 42 following treatment with AL and CQ (includes any species of infection) (Qu. B)

If there are enough non-vivax episodes, the incidence risk of any recurrent parasitaemia over 28 and 42 days will be assessed by survival analysis (Kaplan Meier). In this analysis patients with any recurrent parasitaemia before day 28/42 are considered failures, patients lost to follow up before day 28/42 are censored at the respective day. Patients with no recurrence until day 28/42 are considered cured for the purpose of this analysis. The comparison of risks between CQ vs AL will inform overall schizontocidal efficacy and post treatment prophylaxis.

6.4.1.4 Proportion of patients with *P. vivax* parasitaemia on day 1, 2 and 3 after treatment with AL, CQ, AL+PQ and CQ+AL (Qu. C)

Parasite clearance will be presented using the proportions of patients who remain parasitaemic on each day (days 1 to 3).

6.4.1.5 Proportion of patients with fever on day 1, 2 and 3 after treatment with AL , CQ, AL+PQ and CQ+PQ (Qu. C)

Fever clearance time will be presented as the proportion of patients who were febrile or had a history of fever on day 0 and who became febrile (<37.5°C) day 1, 2 and 3 with no subsequent measured fever or history of fever on subsequent daily within the next 48 hours. Similar rules as for the parasite clearance will be used to handle missing visits.

6.4.2 Radical Cure Endpoints:

6.4.2.1 Incidence rate of all recurrent episodes of *P. vivax* parasitaemia over one year (Qu. D &E)

The total number of *P. vivax* episodes occurring during the 1 year follow up will be presented as incidence estimate in person year of observation (PYO) per treatment arm.

Incidence rates will be calculated by dividing the number of *P. vivax* episodes by the number of person-years of observation (PYO) in the study population. On the individual patient level the start date for PYO is the day of enrolment into the study, stop date is the last visit performed (either completed study at 1 year or any last visit before lost to follow up and/or censoring). The period between start and stop dates for each patient will be calculated in days and divided by 365 to determine PYO, which will then be summed for all participants.

Patients receiving antimalarial treatment will be assumed to have a period of 28 days of post treatment prophylaxis in the chloroquine arm and 14 days in the AL arm following each antimalarial treatment and this period will thus be subtracted from their total period of follow up (time at risk). Patients who received Quinine will have 7 days subtracted.

For this analysis an intention to treat approach is used as the primary approach. Patients who received a treatment other than allocated at enrollment for other episodes with *P. vivax* mono-infections are excluded from this time point in the mITT analyses. Comparison between incidence rates will be presented as Incidence Rate Ratios (IRR) calculated using a negative binomial regression model.

The comparison of risks between CQ vs CQ+PQ and AL vs AL+PQ will inform primaquine efficacy (Qu. D) and the comparison of risks between CQ+PQ vs AL+PQ will inform overall efficacy for radical cure (Qu. E).

In a sensitivity analysis the incidence will be recalculated without subtracting the period of post treatment prophylaxis, to quantify the total incidence rate of all recurrences.

6.4.2.2 The cumulative risk of recurrent *P. vivax* parasitaemia over one year (Qu. D &E)

The incidence risk of recurrent *P. vivax* over total follow up (up to 12 months) will be assessed by survival analysis (Kaplan Meier). In this analysis patients with a recurrent vivax parasitaemia before month 12 are considered failures, patients lost to follow up or with other a non-vivax parasitaemia before month 12 are censored at the respective day. Patients with no recurrence until month 12 are considered cured for the purpose of this analysis.

The comparison of risks between CQ vs CQ+PQ and AL vs AL+PQ will inform primaquine efficacy (Qu D) and the comparison of risks between CQ+PQ vs AL+PQ will inform overall efficacy for radical cure (Qu E).

6.4.2.3 Incidence rate of any parasitaemia at 1 year following treatment (includes any species of infection) (Qu. D & E)

If there are enough non-vivax episodes the analyses will be performed similar as outlined in 6.4.2.1 but for any parasitaemia irrespective of species. In this analysis, patients with any recurrent parasitaemia before month 12 are considered failures, patients lost to follow up before month 12 are censored at the respective day. Patients with no recurrence until month 12 are considered cured for the purpose of this analysis.

6.4.3 Effectiveness endpoints

For the effectivness analysis the enrollment episode (semi-supervised)) will be compared to the first recurrence (unuspervised) This analysis will only be conducted if a sufficient number of cases with recurrences are available. The incidence risk of recurrent *P. vivax* parasitaemia at 12 months following unsupervised treatment (Qu. F)

The cumulative risk of recurrent *P. vivax* after unsupervised treatment at 1 year will be assessed by survival analysis (Kaplan Meier). The starting point of the survival analysis will be the day of the first vivax recurrence. Only patients with a recurrence will therefore be included in this analysis. Patients with a recurrent vivax parasitaemia thereafter and before month 12 are considered failures, patients lost to follow up or with another non-vivax parasitaemia before month 12 are censored at the respective day. Patients with no recurrence until month 12 are considered cured for the purpose of this analysis. Comparison will be made within treatment arms with risk of recurrence after supervised treatment (6.4.2.2).

A similar subgroup analyses will be performed to compare patients who received a full dose and those who received an incomplete dose due to missed visit or not adherence on the days between supervised treatment days.

6.5 Analyses of safety data

The summary statistics (including number, mean, median, interquartile range, standard deviation, minimum and maximum) of all vital signs variables (values and changes from baseline) will be calculated for each visit by treatment group.

6.5.1 Proportion of patients completing 3 days of schizontocidal treatment

The proportion of patients who completed the 3 day schizontocidal treatment will be presented as an indicator for tolerability of the treatment.

6.5.2 Proportion of patients completing 14 days of primaquine treatment in the two primaquine treatment arms

The proportion of patients who completed the 14 day primaquine treatment will be presented as an indicator for tolerability of the treatment.

6.5.3 Dose Vomiting

Initial tolerability is an important measure of acute tolerability. The proportion of patients vomiting their medication within 1 hour of administration will be recorded on each day of CQ or AL drug administration. In addition the proportion of patients vomiting any of their primaquine doses during the 14 day course, will be reported and stratified by vomiting doses 1-3, vomiting doses 4-7 and vomiting in doses 8 to 14.

6.5.4 Analysis of Adverse Events and Severe Adverse Events

Malaria should not be reported as an AE since it is an efficacy outcome and should be reported as such. The following summaries will be present by treatment group:

- description of all deaths during the 1 year follow up period
- the total number of patients with any SAE during the study period
- the total number of patients with any AE during the study period

Statistical tests of association, using logistic regression between treatment group and occurrence of AE/SAE will be performed for the number (%) of patients with any AE or SAE.

6.5.5 Haematological Recovery

Mean Hb levels at Day 0, 3, 7, 14 and 28 will be presented per treatment arm. In addition, the mean (SD) change in hemoglobin results will be presented by treatment arm for the respective periodsThe fractional fall in Hb between baseline and days 3, 7, 14 and day 28, will be calculated, as well as the proportion of patients with anaemia less than 7g/dl between day 3 and day 7.

6.6 Analyses of pharmacokinetic data (drug concentrations)

6.6.1 CQ concentration at day of recurrence

For patients with recurrent parasitaemia before day 42 the mean (95%CI) drug concentration at the day of recurrence will be presented. The proportion of patients with recurrences that present with any CQ concentration and those without CQ in their peripheral blood will be tabulated. Further the proportion of patients with concentration ≥100ug/ml and the proportion of patients <100ug/ml will be presented. Patients with adequate drug concentrations at the day of recurrence are indicative of CQ resistant parasites.
